# Use of an Integrated Multi-Omics Approach To Identify Molecular Mechanisms and Critical Factors Involved in the Pathogenesis of *Leptospira*

**DOI:** 10.1128/spectrum.03135-22

**Published:** 2023-02-28

**Authors:** Sridhar Kavela, Pallavi Vyas, Jusail CP, Sandeep K. Kushwaha, Subeer S. Majumdar, Syed M. Faisal

**Affiliations:** a Laboratory of Vaccine Immunology, National Institute of Animal Biotechnology, Hyderabad, India; b Regional Centre for Biotechnology, Faridabad, India; c Bioinformatics Lab, National Institute of Animal Biotechnology, Hyderabad, India; d Gene and Protein Engineering Lab, National Institute of Animal Biotechnology, Hyderabad, India; Forschungszentrum Jülich GmbH

**Keywords:** transcriptomics, proteomics, *Leptospira*, pathogenesis, host-pathogen interaction

## Abstract

Leptospirosis, a bacterial zoonosis caused by pathogenic *Leptospira* spp., is prevalent worldwide and has become a serious threat in recent years. Limited understanding of *Leptospira* pathogenesis and host response has hampered the development of effective vaccine and diagnostics. Although *Leptospira* is phagocytosed by innate immune cells, it resists its destruction, and the evading mechanism involved is unclear. In the present study, we used an integrative multi-omics approach to identify the critical molecular factors of *Leptospira* involved in pathogenesis during interaction with human macrophages. Transcriptomic and proteomic analyses were performed at 24 h postinfection of human macrophages (phorbol-12-myristate-13-acetate differentiated THP-1 cells) with the pathogenic Leptospira interrogans serovar Icterohaemorrhagiae strain RGA (LEPIRGA). Our results identified a total of 1,528 transcripts and 871 proteins that were significantly expressed with an adjusted *P* value of <0.05. The correlations between the transcriptomic and proteomic data were above average (*r* = 0.844), suggesting the role of the posttranscriptional processes during host interaction. The conjoint analysis revealed the expression of several virulence-associated proteins such as adhesins, invasins, and secretory and chemotaxis proteins that might be involved in various processes of attachment and invasion and as effectors during pathogenesis in the host. Further, the interaction of bacteria with the host cell (macrophages) was a major factor in the differential expression of these proteins. Finally, eight common differentially expressed RNA-protein pairs, predicted as virulent, outer membrane/extracellular proteins were validated by quantitative PCR. This is the first report using integrated multi-omics approach to identify critical factors involved in *Leptospira* pathogenesis. Validation of these critical factors may lead to the identification of target antigens for the development of improved diagnostics and vaccines against leptospirosis.

**IMPORTANCE** Leptospirosis is a zoonotic disease of global importance. It is caused by a Gram-negative bacterial spirochete of the genus *Leptospira*. The current challenge is to detect the infection at early stage for treatment or to develop potent vaccines that can induce cross-protection against various pathogenic serovars. Understanding host-pathogen interactions is important to identify the critical factors involved in pathogenesis and host defense for developing improved vaccines and diagnostics. Utilizing an integrated multi-omics approach, our study provides important insight into the interaction of *Leptospira* with human macrophages and identifies a few critical factors (such as virulence-associated proteins) involved in pathogenesis. These factors can be exploited for the development of novel tools for the detection, treatment, or prevention of leptospirosis.

## INTRODUCTION

Leptospirosis, a neglected zoonotic disease, is prevalent worldwide and is of significant importance. Global human leptospirosis incidences are increasing, with an estimated 1.03 million cases annually, of which almost 60,000 are fatal. The actual disease burden of leptospirosis may be more than the estimated. The disease is caused by pathogenic *Leptospira* spp. that enter the susceptible host through a skin aberration or a mucosal route and disseminate via blood to multiple tissues. Disease symptoms range from asymptomatic to the exhibition of flu-like symptoms and multiorgan failure in severe cases. The currently available diagnosis is based on the detection of antibodies by microscopic agglutination test (MAT) or enzyme-linked immunosorbent assay that could only be detected at a late stage of infection. The major challenge here is early detection of the infection since antibiotic treatment is not very effective once the bacteria enter the vital organs and cause significant damage. The current killed vaccine used mainly in animals induces serovar-specific short-term immunity and does not provide cross-protection. Hence, understanding *Leptospira* host interaction to identify the critical factors involved in pathogenesis is very important for developing improved vaccines and diagnostics.

To identify such critical factors (virulence factors), previous studies used several conditions that mimic the *in vivo* host environment, such as temperature, osmolarity, iron starvation, and the presence of serum (1–6). To overcome the limitations of *in vitro* mimicking of the host condition, bacteria were cultivated in a dialysis membrane chamber implanted within the peritoneal cavities of rats in order to characterize leptospires in a truly mammalian host-adapted state (7). Although these *in vitro*-mimicking and host-adapted conditions influenced *Leptospira* behavior in terms of the modulation of various virulence factors, they could not provide insight into changes that occur when bacteria interact with the innate immune cells, particularly phagocytic cells such as macrophages (8–10). Macrophages, as professional phagocyte cells, have evolved and manifested to eliminate pathogenic bacteria. Although pathogenic *Leptospira* is not considered a typical intracellular pathogen, several studies have shown that it can attach to, invade, and induce the apoptosis of mammalian macrophages to escape an innate response during the early stage of infection (11, 12). A previous study has demonstrated the differential survivability of L. interrogans within murine and human macrophages; however, a recent study has shown that the bacteria exit both types of macrophages without intracellular replication (13, 14). The uptake of L. interrogans by phagocytes was also demonstrated by the zebrafish embryo model, which suggests phagocytosis as a critical defense during early stages of infection (15).

During the last decade, an omics has emerged as an effective tool in basic, translational, and clinical research for the study of biological pathways involved in pathogen replication, host response, and disease progression during host-pathogen interactions (16). Various *in vitro* macrophage infection studies have been carried at proteomics and transcriptomic level in several pathogenic bacterial species, such as Salmonella enterica serovar Typhimurium (17–19), enteropathogenic Escherichia coli (20), Mycobacterium tuberculosis (21), and Bordetella pertussis (22). These studies have revealed the various virulence factors and bacterial cellular responses against host cells. In order to identify such factors, previous studies focused on examining the differential host immune response to various sources of bacterial virulence in a mouse macrophage cell line (J774A.1) infected with pathogenic *Leptospira* (23). The transcriptional response of *Leptospira* during its interaction with macrophages revealed the downregulation of several outer membrane proteins (OMPs) and associated transcription factors, which could indicate a host immune evasion strategy (24). Despite various studies on *Leptospira*-host interactions, there is a limited understanding of the mechanism of host infectivity and pathogenesis (25, 26).

**FIG 1:**
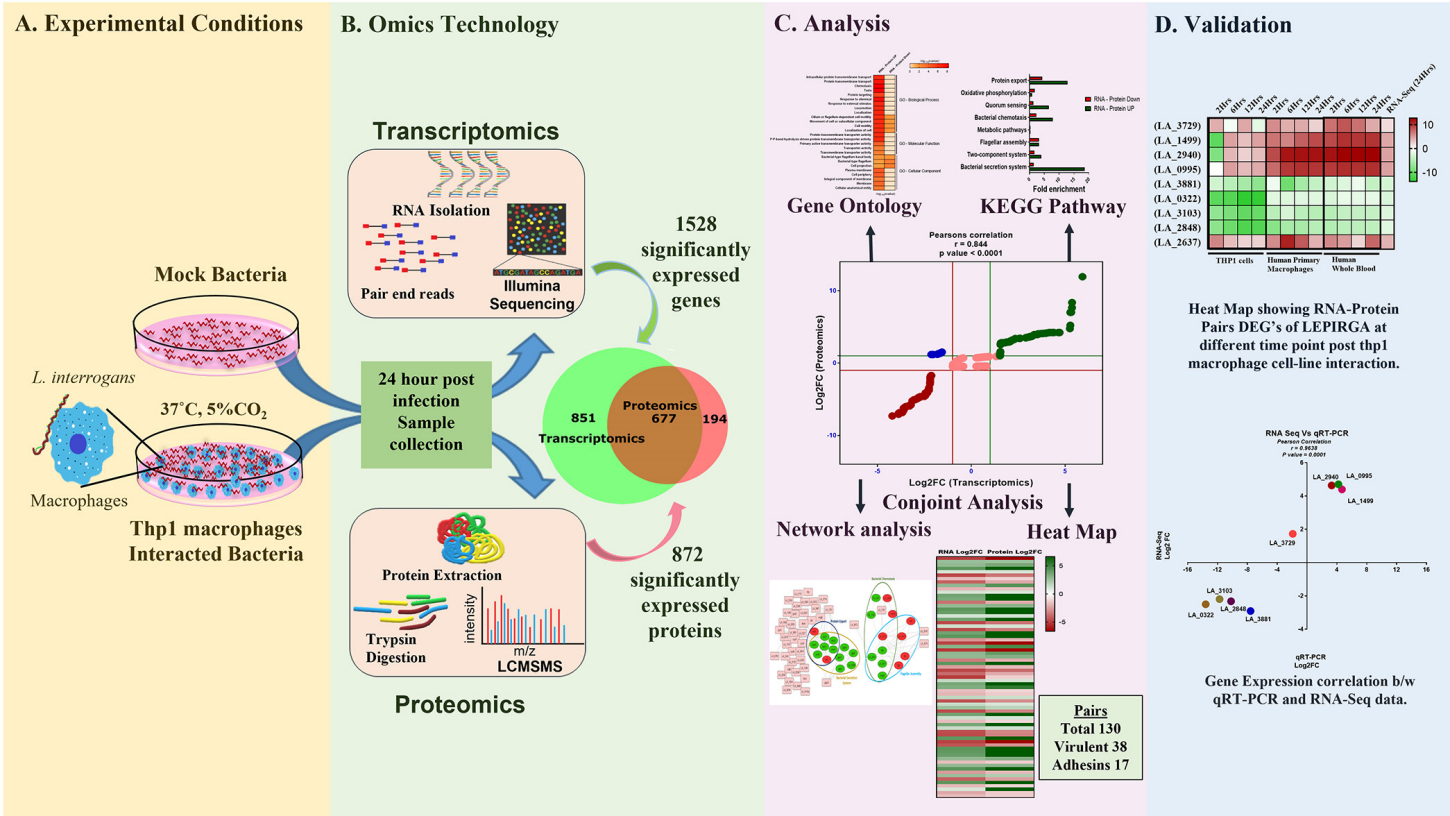
Schematic representation of identifying critical factors of pathogenic *Leptospira* upon interaction with human macrophages by integrating transcriptomics and proteomics techniques. (A) *In vitro* macrophage infection model. (B) Integration of transcriptome and proteome data. (C) Bioinformatics analysis. (D) Validation of OMICs data.

Here we used multi-omics (transcriptomics and proteomics) approach to understand Leptospira pathogenesis during interaction with human macrophages. PMA (phorbol 12-myristate 13-acetate) differentiated THP-1 cells were infected with virulent *Leptospira interrogans* serovar Icterohaemorrhagiae str. RGA (*LEPIRGA*) and at 24hrs post infection, whole transcriptome (Next-generation RNA-Sequencing) and proteome analysis was done. The transcriptome and proteome data obtained were integrated, and critical factors involved in pathogenesis were filtered using various bioinformatics tools. The expression of selected critical factors was then validated by quantitative reverse transcription-PCR (qRT-PCR) (Fig. 1).

## RESULTS

### Global transcriptional profiling of *Leptospira* upon interaction with human macrophages.

**FIG 2:**
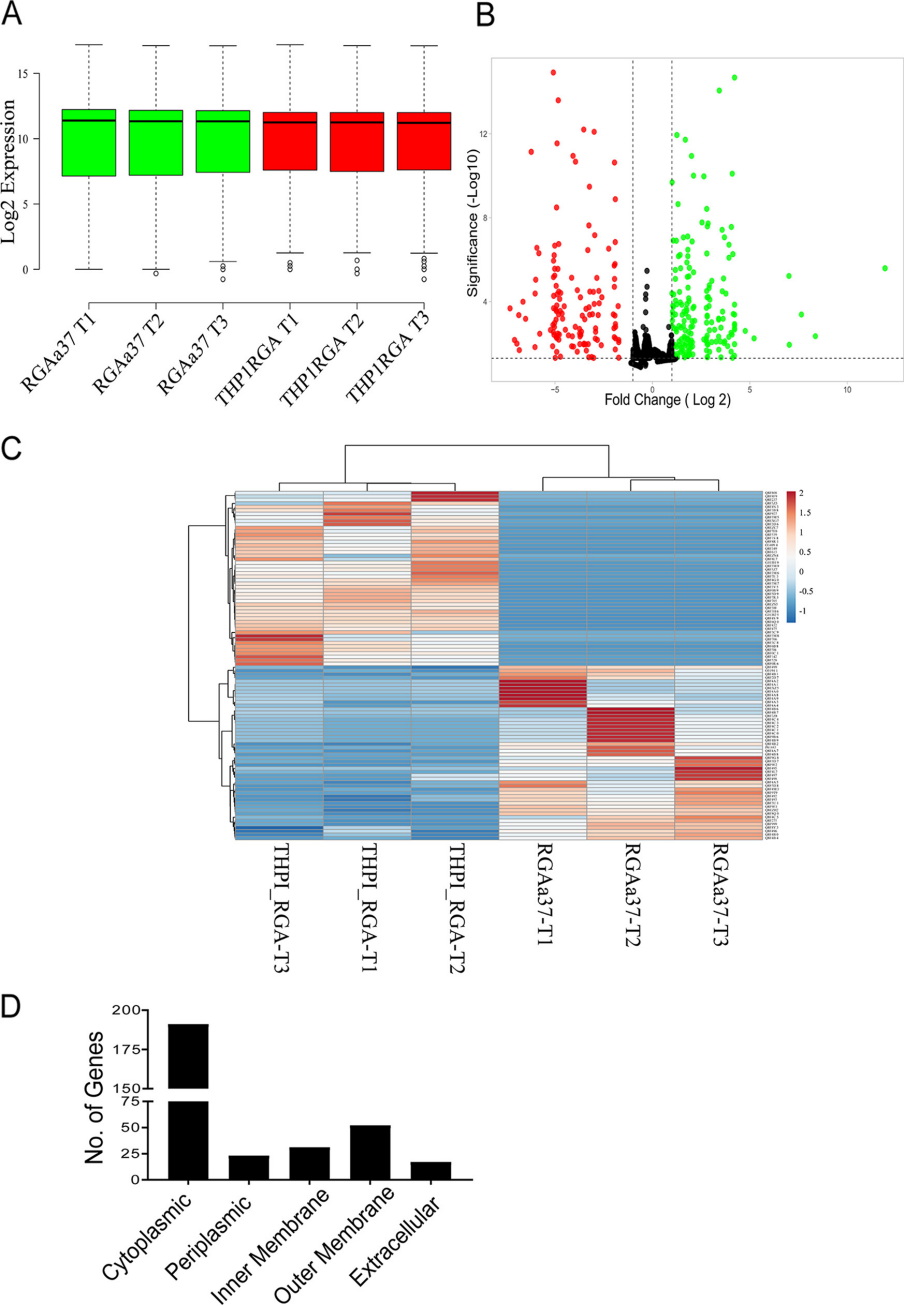
Transcriptome analysis of *Leptospira* during interaction with human macrophages. (A) Box plot of logCPM expression values across the samples. (B) Volcano plot representation of differential expression analysis of *Leptospira* gene in bacterial mock samples and host adopted bacterial samples. Genes with a significance (*P*_adj_) of ≤0.05 and genes with a significance (*P*_adj_) of ≤0.05 and a log_2_-fold change greater than 1 are represented in red and green, respectively. (C) Heatmap of most variable genes across the samples (D) Subcellular localization of DEGs.

The test and control samples were sequenced. In total, 127 million read pairs (2 × 150 bp) were generated from the sequencing, and an average of 21 million reads was found per sample. After quality control, an average of 15 million high-quality reads of an average length of 151 bp were used for transcriptome assemblies (see Table S1 in the supplemental material). High-quality reads of each sample were aligned over LEPIN transcriptome assembly using Bowtie 2 with default parameters to quantify the transcriptomic variations among samples. FeatureCount software was used to quantify the transcript abundance. A box plot was plotted to compare the sample level distributions of gene expression data postnormalization for six samples (three control [green] and three test [host-interacted, red] samples) (Fig. 2A). In all, 2,156 differentially expressed *Leptospira* genes were identified using DESeq2 software, 1,528 of which were found to be significant at an adjusted *P* value (*P*_adj_) of ≤0.05. Among the significant genes, 314 were differentially expressed with a log_2_ fold change (Log_2_FC) ≥ +1 and ≤ −1 (Fig. 2B; see also Table S2). Correlation based clustered heat map depicting relations of the top 50 differential expressed genes (DEGs) across the samples (Fig. 2C; see also Table S3). When the 314 DEGs were subjected to subcellular localization, we found that most of the genes are localized to cytoplasm, followed by the outer membrane (Fig. 2D).

### Liquid chromatography-tandem mass spectrometry-based identification of proteins that are modulated during *Leptospira* macrophage interaction.

**FIG 3:**
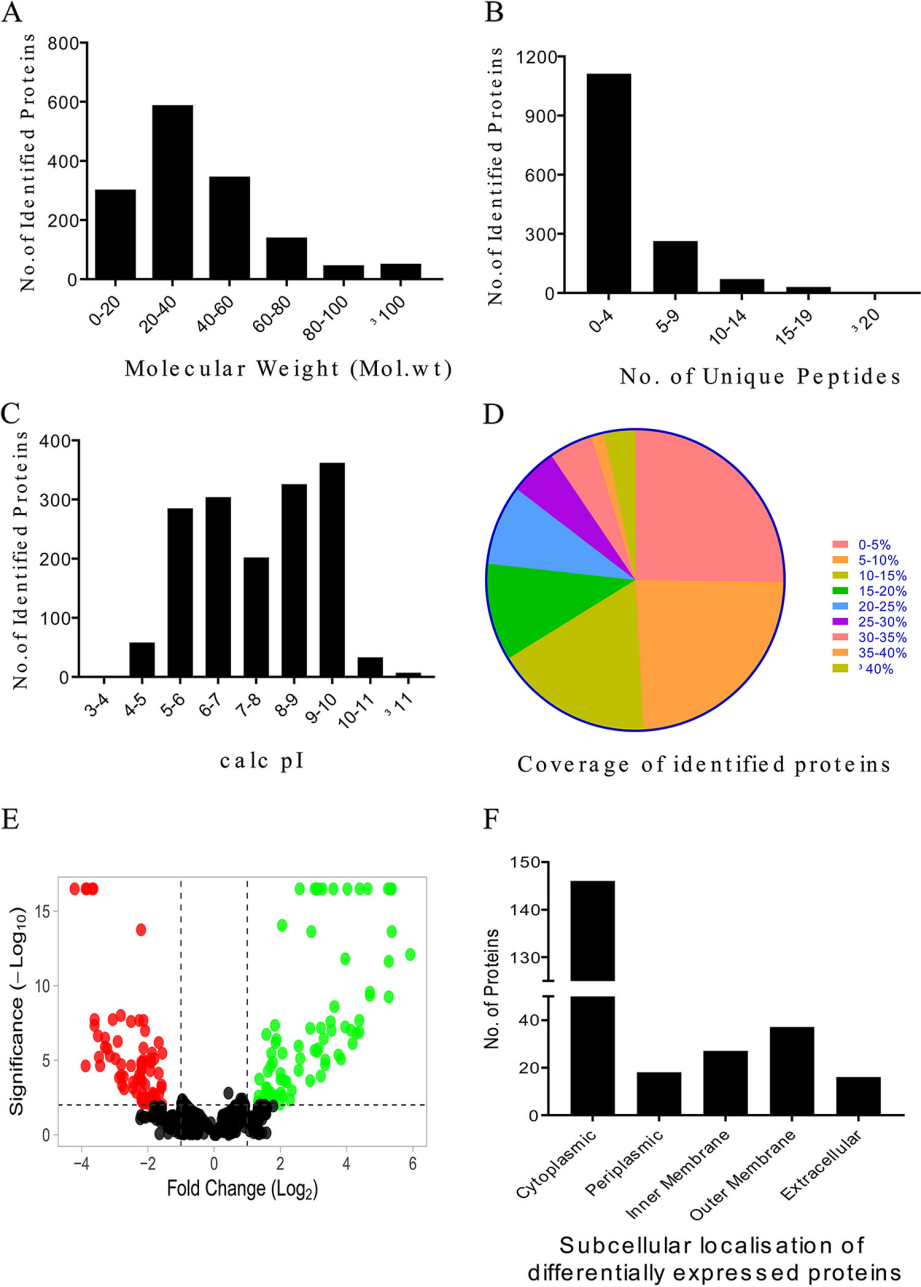
Dynamic profiling of the proteome of *Leptospira* during interaction with human macrophages. (A to D) Distributions of the molecular weight (A), number of unique peptides (B), calculated pI (C), and sequence coverage (D) of the identified proteins. (E) Volcano plots displaying proteins changing in leptospiral protein abundance at 24 h after host adaptation. Proteins with a Log_2_FC of >1 are in the green area. Proteins with a Log_2_FC of <−1 are in the red area. Proteins with a *P* value of <0.05 or a –log_10_(*P* value) of >1.3 are indicated above the horizontal black dotted line. (F) Subcellular localization of proteins that are dysregulated in *Leptospira* during the host adaptation.

In proteome data analysis, 1,152 proteins were identified, and three or more unique peptides covered 75.23% of the identified proteins (Fig. 3A). Among the identified proteins, 83.83% had molecular weights in the range of 10 to 60 kDa (Fig. 3B), and 96.25% had pI values ranging from 4 to 10 (Fig. 3C). In addition, 50.88% of the identified proteins showed more than 10% sequence coverage, while 23.3% had sequence coverage above 20% (Fig. 3D). A volcano plot was also generated to illustrate further differences in LEPIRGA protein expression and 244 proteins with *P* values ≤ 0.05 and Log_2_FC ≥ +1 and ≤ −1 were significantly up- and downregulated (Fig. 3E; see also Table S4). The top 50 list of differentially regulated proteins is summarized in Table S5. All 244 differentially regulated proteins have been explored for subcellular localization using the CELLO v.2.5 webserver. The majority of the LEPIRGA proteins, which are differentially regulated belong to cytoplasmic (61.26%), outer membrane (17.03%), periplasmic (6.31%), inner membrane (10.16%), and extracellular (5.21%) classes. (Fig. 3F). Gene ontology enrichment analysis and KEGG pathway analysis were also performed to predict the functions and to identify the significant pathways enriched among these differentially expressed proteins (DEPs). The most significantly enriched GO biological terms were cellular process, chemotaxis, localization, locomotion, transport, and response to chemicals. Catalytic activity, binding, intramolecular transferase activity, and protein transmembrane transporter activity were the most significantly enriched GO terms for molecular function. In addition, the GO terms for cellular components were primarily enriched in the cellular anatomical entity, cytoplasm, intracellular, cell periphery, and bacterial-type flagellum (see Fig. S2A). KEGG pathway analysis of differentially regulated proteins revealed that most of the proteins were mainly involved in bacterial secretory system, protein export, bacterial chemotaxis, flagellar assembly, quorum sensing, metabolic pathways, and two-component systems (see Fig. S2B).

### Integration of transcriptomics and proteomics data to identify the critical factors involved in pathogenesis.

**FIG 4:**
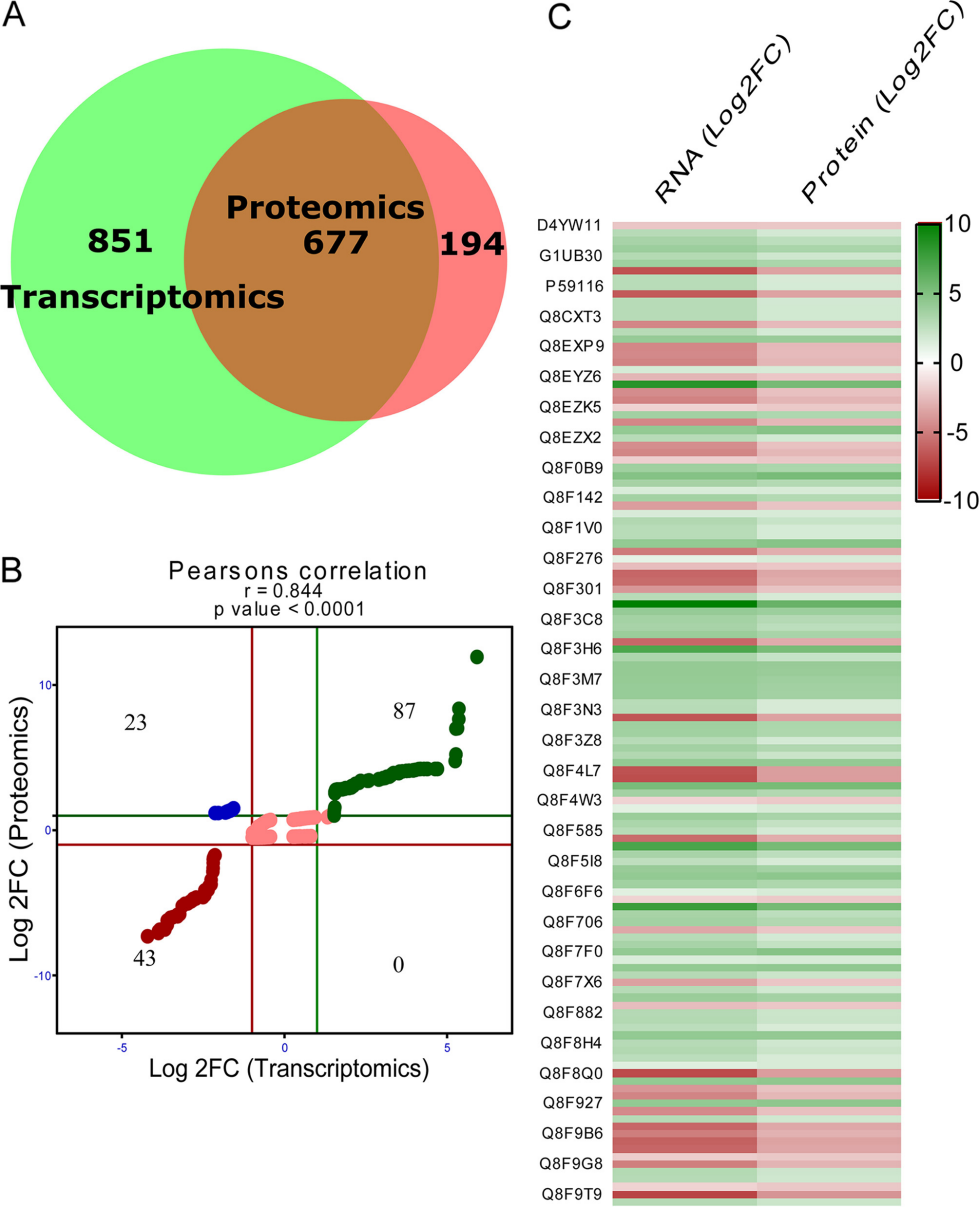
Correlation and conjoint analysis of transcriptome and proteome. (A) Venn diagram displaying the common and the unique genes found at transcriptome and proteome level. (B) Comparisons of the expression ratios from transcriptomic (*y* axis) and proteomic (*x* axis) profiling. Log_2_FC expression ratios are calculated as the mRNA or protein changes in *Leptospira* at 24 h after host adaptation. Significant changes in expression are indicated as follows: quadrants 2 and 8, proteins only; quadrants 4 and 6, transcripts only; quadrant 3, green; and quadrant 7, red. Both red and green lines represent an mRNA fold change of ±1 and a protein fold change of ±1. (C) Heat maps representing the changes in the abundance of the genes in both quadrants 3 and 7 during L. interrogans Icterohaemorrhagiae interaction with human macrophage Thp1 cells.

**FIG 5:**
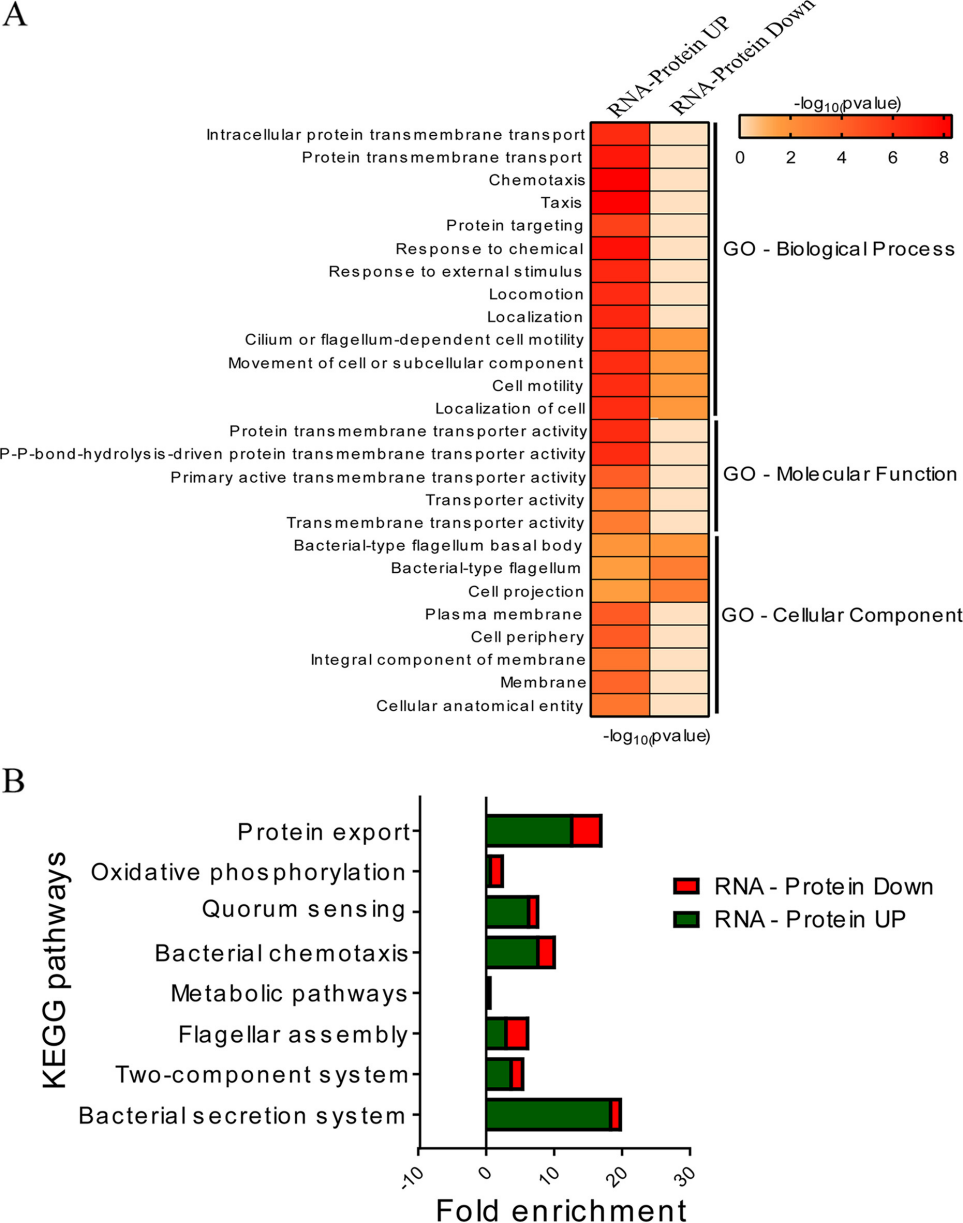
Gene ontology and KEGG pathway analysis of RNA-protein pairs. (A) The functional classification of the common 130 transcript-protein pairs DEGs was analyzed by GO enrichment analysis. (B) KEGG pathway analysis was performed to investigate the significant pathways enriched by the 130 common differentially expressed genes in transcriptomes and proteomes.

We integrated the transcriptomics and proteomics data and performed conjoint analysis to understand how transcriptional processes lead to proteomic changes during *Leptospira*-macrophage interaction. A total of 677 genes were found common in both data sets (Fig. 4A). A scatterplot analysis of the log_2_ transformation was performed to display the distribution of the corresponding transcript/protein ratios (Fig. 4B). We found 87 upregulated (quadrant 3) and 43 downregulated (quadrant 7) genes with consistent trends at both protein and transcript levels. We also identified 23 genes (quadrant 1) that were only regulated at the posttranscriptional level and 524 genes (quadrant 5) that were regulated at both transcriptional and posttranscriptional levels. The changes in the abundances of 130 common DEGs and DEPs are represented as a heat map (Fig. 4C). Gene ontology enrichment analysis was performed further to predict the functions of these 130 common DEGs and DEPs. The most significantly enriched GO biological terms belong to the cell motility, protein targeting, chemotaxis, locomotion, localization, and response to external stimulus. Protein transmembrane and P-P bond hydrolysis-driven protein transmembrane transporter activity were the most significantly enriched GO terms for molecular function. In addition, the GO terms for cellular components were primarily enriched in the cellular anatomical entity, bacterial-type flagellum, cell projection, and integral component of membrane (Fig. 5A). KEGG pathway analysis was also performed to identify the significant pathways enriched among common DEGs and DEPs. We found that the most enriched KEGG pathways were mainly metabolic pathways, bacterial secretion system, chemotaxis, flagellar assembly, two-component system, and quorum sensing (Fig. 5B). The 130 common DEGs and DEPs were further categorized into groups based on virulence, adhesins, invasins (27), secretome (28), and subcellular location using *in silico*-based analysis (Table 1). Of 130 proteins, 65 were cytoplasmic, including 20 virulent proteins, 3 adhesins, and 1 protein that was both virulent and adhesin (LA_1004, putative lipoprotein). Fifteen belonging to the inner membrane include three virulent proteins (FliQ, DltB, and GspF). Of 14 periplasmic proteins, 7 were virulent, 1 was adhesin, and 2 were both virulent and adhesin (Lipl21 and OmpA). Twenty-three were predicted to be OMPs, of which thirteen were virulent and nonadhesin proteins and three were both virulent and adhesin (LA_3913, LA_2940, and LA_0995). Thirteen were extracellular, of which eleven were virulent and adhesins, and two were nonadhesin virulent (Table 1; see also Fig. S4A and B). We also performed a protein-protein interaction (PPI) network using a STRING database and network analysis by using the Cytoscape plugin for the 130 common differentially expressed RNA-protein pairs. Network statistics obtained by using the STRING database revealed that the extracted interactome consisted of 130 nodes and 162 edges. The average node degree and average local clustering coefficient of the network were determined to be 2.49 and 0.361, respectively. Manual curation of the PPI network data revealed that proteins that are involved in bacterial secretion system, flagellar assembly, protein export, and chemotaxis are highly connected, with each member having five or more interacting partners (see Fig. S4C). Moreover, the network analysis estimated several other topological parameters, such as the average number of neighbors, the network diameter, the network radius, the characteristic path length, the cluster coefficient, the network density, and the network heterogeneity (see Table S6). Since, in the PPI network, hub proteins represent highly connected nodes with important biological properties, we used the Cytohubba tool to explore these proteins in 130 common differentially expressed RNA-protein pairs. Using a radiality and betweenness algorithm, we determined the top 20% hub proteins (see Table S7). CapA (LA_1351) was the top scorer hub protein by both methods and is predicted to be virulent and is upregulated upon host cell interaction. The most common hub proteins identified by both algorithms belong to bacterial secretory systems and protein export.

**TABLE 1:** Categorization of *Leptospira* commonly expressed genes based on their biological localization and *in silico* analysis

Category and ID no.	Protein name(s)	Gene name(s)	Log_2_FC		Adhesin	Pathogenic	Invasion	Secretory
			RNA	Protein				
Cytoplasmic								
D4YW11	Uncharacterized protein	LA_4314a	−2.24	−2.19	−	−	−	−
D4YW40	PINc domain-containing protein	LA_1816a	2.81	1.70	−	+	−	−
P61443	Chaperone protein DnaK (HSP70, heat shock 70-kDa protein, heat shock protein 70)	*dnaK*, LA_3705	−6.51	−3.61	−	−	−	+
Q8EX79	4*a*-Hydroxytetrahydrobiopterin dehydratase (EC 4.2.1.96)	LB_335	−4.71	−2.73	−	−	−	−
Q8EXD0	TonB protein	LB_283	2.79	1.62	−	−	−	−
Q8EXP9	Precorrin-2 C-20 methyltransferase	*cobF*, LB_159	−4.72	−2.78	−	−	−	−
Q8EXZ5	Antagonist of anti-sigma factor	LB_062	−4.89	−2.87	−	−	−	−
Q8EY75	Uncharacterized protein	LA_4342	1.57	1.55	−	−	−	−
Q8EYZ6	Two-component response regulator	LA_4065	−2.85	−2.20	−	−	−	−
Q8EZC7	Acyl-CoA dehydrogenase	*caiA*, LA_3928	8.36	5.36	−	−	−	−
Q8F089	MaoC-like acyl dehydratase	*maoC*, LA_3607	−4.76	−2.81	−	−	−	+
Q8F0B8	Flagellar FlbD family protein	LA_3578	−1.91	−2.18	−	−	−	−
Q8F0C1	Flagellar protein FliL	*fliL*, LA_3575	4.77	5.25	−	+	−	−
Q8F0R6	Protein translocase subunit SecE	*secE*, LA_3426	3.48	2.90	−	−	−	−
Q8F0S0	50S ribosomal protein L10	*rplJ*, LA_3422	1.47	1.54	−	−	−	−
Q8F142	Uridylate kinase (UK, EC 2.7.4.22, uridine monophosphate kinase, UMP kinase, UMPK)	*pyrH*, LA_3296	3.52	2.90	−	−	−	−
Q8F1P9	Adenylosuccinate lyase (ASL, EC 4.3.2.2, adenylosuccinase)	*purB*, LA_3080	1.53	1.54	−	−	−	−
Q8F275	M23 family metalloendopeptidase	*nlpD*, LA_2901	−5.23	−3.15	−	−	−	−
Q8F2D7	Adenylate/guanylate cyclase	*cyaA*, LA_2834	−6.02	−3.47	−	−	−	−
Q8F339	TPR-repeat-containing protein	*pilF*, LA_2572	2.78	1.58	−	+	−	−
Q8F349	Glycosylhydrolase	*bglX*, LA_2561	11.94	5.92	−	−	−	−
Q8F3C8	Hypothetical lipoprotein	LA_2478	3.52	2.93	−	+	−	−
Q8F3C9	TfoX-like protein	*tfoX*, LA_2477	3.40	2.55	−	−	−	−
Q8F3D6	Putative lipoprotein	LA_2470	3.93	3.36	−	+	−	−
Q8F3D7	Inhibitor of MCP methylation	LA_2469	−5.93	−3.29	−	−	−	−
Q8F3H6	Chemotaxis protein CheW	*cheW*, LA_2427	7.01	5.27	−	−	−	−
Q8F3I0	Chemotaxis response regulator CheY	LA_2423	3.19	2.18	−	+	−	−
Q8F3M7	Protein-secreting ATPase (EC 7.4.2.8)	*gspE*, LA_2374	4.09	3.76	−	+	−	−
Q8F3N2	Type II secretion system protein J	LA_2369	2.78	1.58	−	+	−	−
Q8F3U1	Long-chain fatty acid CoA ligase	*faa1*, LA_2309	−6.64	−3.64	−	−	−	−
Q8F3Z8	Endonuclease	LA_2250	2.81	1.62	−	+	−	−
Q8F432	Endoflagellar motor protein B	*motB*, LA_2215	3.60	3.09	−	+	−	−
Q8F4G0	Flagellar motor switch protein FliM	*fliM*, LA_2081	4.20	4.13	−	+	−	−
Q8F4M3	Flagellin	LA_2017	−6.96	−3.88	−	+	−	−
Q8F4N3	Flagellar motor switch protein FliG	*fliG*, LA_2007	5.22	5.27	−	+	−	−
Q8F4S9	Protein translocase subunit SecA (EC 7.4.2.8)	*secA*, LA_1960	3.59	3.04	−	−	−	−
Q8F527	Histidine kinase sensor protein	LA_1860	1.08	1.53	−	−	−	−
Q8F539	ISLbp1 transposase	LA_1848	3.27	2.29	−	−	−	−
Q8F585	PINc domain-containing protein	LA_1799	2.79	1.62	+	−	−	−
Q8F5D8	Putative protein-glutamate methylesterase/protein-glutamine glutaminase (EC 3.1.1.61, EC 3.5.1.44)	*cheB2*, LA_1744	−5.78	−3.22	−	−	−	−
Q8F5D9	Methylase of chemotaxis methyl-accepting protein	*cheR*, LA_1743	7.03	5.32	−	−	−	−
Q8F5I8	Signal transduction histidine kinase	*baeS*, LA_1693	2.54	1.55	−	+	−	−
Q8F5Z7	RNA-binding protein Hfq	*hfq*, LA_1517	4.04	3.54	−	−	−	+
Q8F6Q0	Chemotaxis protein CheA (EC 2.7.13.3)	*cheA*, LA_1251	7.65	5.36	−	−	−	−
Q8F7C7	NifU_N domain-containing protein	LA_1019	−3.42	−2.26	−	+	−	−
Q8F7E1	Putative lipoprotein	LA_1004	2.81	1.71	+	+	−	−
Q8F7E3	Virulence-associated protein VagC	*vagC*, LA_1002	3.40	2.34	−	−	−	−
Q8F7Q4	NADH-quinone oxidoreductase subunit F (EC 7.1.1.−)	*nuoF*, LA_0890	1.42	1.54	−	−	−	−
Q8F7R5	Uncharacterized protein	LA_0879	4.21	4.29	−	−	−	−
Q8F7X6	Response regulator containing a signal receiver domain and a DNA-binding domain	*citB*, LA_0816	−3.72	−2.26	−	−	−	−
Q8F7X9	PINc domain-containing protein	LA_0813	2.83	1.78	−	+	−	−
Q8F808	TM2 domain-containing protein	LA_0782	3.93	3.51	+	−	−	−
Q8F847	Putative lipoprotein	LA_0712	−2.62	−2.20	−	+	−	−
Q8F8F0	MCE-related protein	LA_0607	2.94	2.05	−	+	+	−
Q8F8F2	SET family protein	LA_0605	2.64	1.56	−	−	−	−
Q8F8F9	Transcriptional regulator	LA_0598	4.21	4.18	−	−	−	−
Q8F8H4	Outer membrane protein TolC	LA_0581	2.86	1.86	−	+	−	−
Q8F8L7	Transcriptional regulator	LA_0538	3.04	2.06	−	−	−	−
Q8F8P2	UDP-3-*O*-[3-hydroxymyristoyl] glucosamine *N*-acyltransferase	*lpxD*, LA_0512	1.25	1.53	−	−	−	−
Q8F8S7	Tdh-like protein	LA_0477	−4.15	−2.45	−	−	−	−
Q8F8Y3	SAM-dependent *O*-methyltransferase	LA_0415	−4.81	−2.82	−	−	−	−
Q8F992	Putative lipoprotein	LA_0303	2.85	1.84	−	+	−	−
Q8F9B6	Uncharacterized protein	LA_0279	−5.07	−3.06	−	−	−	−
Q8F9P0	ACT domain-containing protein	LA_0151	2.86	1.87	+	−	−	−
Q8F9P7	Probable GTP-binding protein EngB	*engB*, LA_0144	−1.76	−2.16	−	−	−	−
Extracellular								
G1UB30	OmpL1	LA_3138	2.90	1.88	+	+	−	+
P59116	Sphingomyelinase C2 (EC 3.1.4.12, sphingomyelin phosphodiesterase 2, SMase 2)	*sph2*, LA_1029	2.77	1.57	−	+	+	+
Q8EZH2	Putative lipoprotein	LA_3881	−4.94	−2.91	+	+	−	+
Q8EZK5	Immunoglobulin-like domain-containing protein	LA_3848	−1.73	−2.13	+	+	−	+
Q8EZS3	LigB-like protein	LA_3778	4.21	4.70	+	+	−	+
Q8EZX2	Fimh-like protein	LA_3729	2.81	1.73	+	+	−	+
Q8F1M9	LenB	LA_3103	−3.85	−2.32	+	+	−	+
Q8F1S8	Hemolysin	LA_3050	3.05	2.12	+	+	+	+
Q8F213	Leucine-rich repeat protein	LA_2964	2.76	1.57	−	+	−	−
Q8F2C6	Flagellar hook protein FlgE	*flgE*, LA_2848	−2.60	−2.20	+	+	−	+
Q8F613	Cytoplasmic membrane protein	LA_1499	4.21	4.40	+	+	−	+
Q8F8Q0	Uncharacterized protein	LA_0505	−7.07	−3.88	+	+	−	+
Q8F974	Fibronectin-binding protein	LA_0322	−4.61	−2.51	+	+	−	+
Inner membrane								
G1UB19	Protein translocase subunit SecY	*secY*, LA_0759	3.40	2.55	−	−	−	−
Q8EXG7	Na^+^/solute symporter	*putP*, LB_245	4.11	3.96	−	−	−	−
Q8F0B9	Endoflagellar motor protein A	*motA*, LA_3577	3.69	3.22	−	−	−	−
Q8F301	Flagellar biosynthetic protein FliQ	*fliQ*, LA_2610	−4.11	−2.35	−	+	−	−
Q8F3B8	Predicted membrane protein involved in d-alaninealginate export/acetyltransferase of MBOAT family	*dltB*, LA_2489	4.10	3.83	−	+	−	−
Q8F3M8	Type II secretory pathway component protein F	*gspF*, LA_2373	4.05	3.63	−	+	−	−
Q8F3Z3	Predicted membrane protein involved in d-alaninealginate export/acetyltransferase of MBOAT family	*dltB*, LA_2256	3.65	3.15	−	−	−	−
Q8F4W3	Sec-independent protein translocase protein TatC	*tatC*, LA_1925	−1.73	−2.14	−	−	−	−
Q8F6B8	Uncharacterized protein	LA_1390	3.68	3.15	−	−	−	−
Q8F6H0	Lipoprotein signal peptidase (EC 3.4.23.36, prolipoprotein signal peptidase, signal peptidase II, SPase II)	*lspA*, LA_1336	−1.73	−2.13	−	−	−	−
Q8F705	Protein-export membrane protein SecF	*secF*, LA_1143	3.43	2.60	−	−	−	−
Q8F706	Protein translocase subunit SecD	*secD*, LA_1142	3.58	3.04	−	−	−	−
Q8F8N0	Putative lipoprotein	LA_0525	2.64	1.55	−	−	−	−
Q8F9F2	Cytochrome *c* oxidase subunit 1 (EC 7.1.1.9)	*cyoB*, LA_0243	−6.22	−3.61	−	−	−	−
Q8F9F3	Cytochrome *c* oxidase subunit 2 (EC 7.1.1.9)	*cyoA*, LA_0242	−6.03	−3.51	−	−	−	−
Outer membrane								
P59115	Sphingomyelinase C 1 (EC 3.1.4.12, sphingomyelin phosphodiesterase 1, SMase 1)	*sph-1*, LA_1027	2.78	1.58	−	+	+	+
Q8CVE2	TolC family protein	LA_3927	2.81	1.76	−	+	−	−
Q8CVE4	TolB-related protein	LA_3067	2.82	1.77	−	+	−	−
Q8CXT3	Predicted phospholipase	LA_1541	2.82	1.78	−	−	+	−
Q8EXV3	Transcriptional regulator	LB_104	−4.64	−2.72	−	−	−	−
Q8EZE1	Putative lipoprotein	LA_3913	−4.53	−2.51	+	+	−	−
Q8EZZ1	Putative transcriptional regulator	LA_3710	−4.47	−2.45	−	−	−	−
Q8F1V0	Leucine-rich repeat containing protein	LA_3028	2.77	1.57	−	+	−	−
Q8F237	Uncharacterized protein	LA_2940	4.21	4.63	+	+	−	+
Q8F276	Uncharacterized protein	LA_2900	1.01	1.53	−	−	−	−
Q8F2Z8	Flagellar motor switch protein FliN	LA_2613	−5.83	−3.29	−	+	−	+
Q8F3M5	Type II secretory pathway component protein C	*gspC*, LA_2376	4.15	4.02	−	+	−	−
Q8F3M6	Type II secretory pathway component protein D	*gspD*, LA_2375	4.13	3.96	−	+	−	−
Q8F3N3	Type II secretion pathway related protein EtpK-like protein	LA_2368	2.79	1.61	−	+	−	−
Q8F475	Lipoprotein with phospholipase D domain	LA_2170	3.04	2.07	−	−	−	−
Q8F4L7	Conserved hypothetical lipoprotein	LA_2024	−6.86	−3.84	−	+	−	+
Q8F7F0	Fibronectin type III domain-containing protein	LA_0995	4.21	4.70	+	+	−	−
Q8F7S2	Microbial collagenase (EC 3.4.24.3)	LA_0872	2.92	2.01	−	+	+	−
Q8F882	Methyl-accepting chemotaxis protein	LA_0676	2.93	2.01	−	+	−	−
Q8F927	Uncharacterized protein	LA_0370	4.21	4.38	−	+	−	+
Q8F9F4	SCO1/SenC/PrrC family protein	LA_0241	−2.01	−2.19	−	−	−	−
Q8F9G8	Uncharacterized protein	LA_0227	−5.04	−2.98	−	+	−	−
Q8F9Y8	Methyl-accepting chemotaxis protein	LA_0049	2.93	2.01	−	−	−	−
Periplasmic								
G1UB25	Outer membrane lipoprotein LipL21	*lipL21*, LA_0011	3.86	3.31	+	+	−	+
O34094	LipL32	*lipL32*, LA_2637	3.77	3.27	−	+	−	+
O51941	Flagellar filament 35-kDa core protein (35-kDa antigen, flagellin class B)	*flaB*, *flaB2*, LA_2019	−6.83	−3.68	−	+	−	−
Q8EZN4	EVE domain-containing protein	LA_3818	3.63	3.11	−	+	−	+
Q8EZS1	Hypothetical lipoprotein	LA_3780	−4.73	−2.81	−	+	−	+
Q8F3M9	Type II secretory pathway component protein G	*gspG*, LA_2372	4.04	3.60	−	+	−	+
Q8F3V5	LipL45	*lipL45*, LA_2295	3.71	3.22	−	−	−	+
Q8F528	Catalase (EC 1.11.1.6)	*katE*, LA_1859	3.92	3.35	−	−	−	+
Q8F5I6	Protein-export membrane protein SecG	*secG*, LA_1695	3.41	2.58	−	−	−	+
Q8F6F6	Capsule biosynthesis protein CapA (poly-gamma-glutamate biosynthesis protein)	LA_1351	1.15	1.53	−	+	−	−
Q8F8R1	Putative lipoprotein	LA_0494	4.21	4.38	−	+	−	+
Q8F999	Zn-dependent alcohol dehydrogenase	*adhP*, LA_0296	−5.99	−3.44	−	−	−	+
Q8F9H3	OmpA family lipoprotein	LA_0222	2.91	1.99	+	+	−	+
Q8F9T9	Uncharacterized protein	LA_0100	−7.31	−4.21	+	−	−	−

### Validation of candidate genes using qRT-PCR at the four LEPIRGA infection time points in a THP-1 cell line, human blood primary macrophages, and human whole blood.

**FIG 6:**
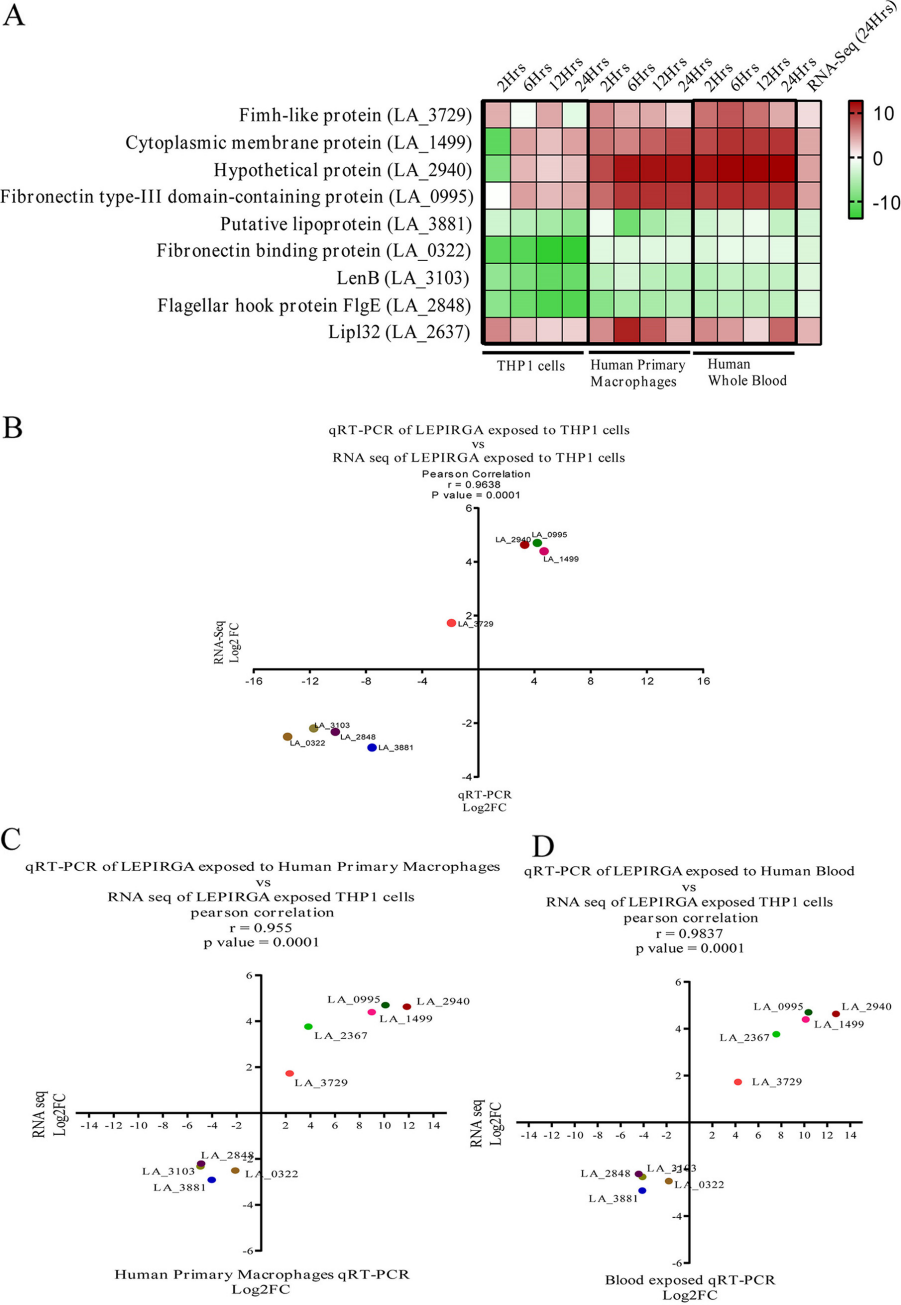
qRT-PCR validation. (A) Heatmap showing the 08 RNA-protein pairs DEGs of LEPIRGA at different times points after interaction with the host (THP-1/HBPM/HWB). (B to D) Gene expression correlation between qRT-PCR (THP-1/HBPM/HWB) and RNA-Seq data.

To verify the accuracy of the RNA sequencing (RNA-Seq) results, eight common DEGs and DEPs (four upregulated and four downregulated) were randomly selected for qRT-PCR verification using specific primers. These DEGs/DEPs were mainly periplasmic, outer membrane, and extracellular (POME) proteins that are predicted to be virulence factors and adhesins (Table 2). A fresh batch of RNA was prepared from LEPIRGA-infected THP-1/HBPM (human blood primary macrophage)/HWB (human whole blood) samples obtained at 2, 6, 12, and 24 h. Expression levels of selected DEGs were analyzed by RT-PCR. LipL32 was used as an experimental/positive control. (Fig. 6A; see also Fig. S5 and Table S8 in the supplemental material). The qRT-PCR result shows that the different expression trends of the validated genes are consistent with the transcriptome results. The qRT-PCR expression values (for LEPIRGA-infected THP-1/HBPM/HWB samples) at 24-h time points of selected DEGs were plotted against the RNA-Seq values of corresponding genes, and high correlation coefficients (*r* = 0.9638/0.955/0.9837) were obtained (Fig. 6B to D). For instance, fibronectin type III domain-containing protein was upregulated in both RNA-Seq data (4.7) and qPCR data (4.2), whereas putative lipoprotein (LA_3881) was downregulated in both RNA-Seq (−2.9) and qPCR (−7.57) analyses (see Table S8).

**TABLE 2:** Common DEGs and DEPs, predicted to be localized to periplasmic, outer membrane, and extracellular locations (POME), that may act as virulence factors and adhesin

Protein ID	Description	Gene name(s)	Log_2_FC		Adhesin	Virulent
			RNA	Protein		
Q8EZS3	LigB-like protein	LA_3778	4.214690149	4.702435914	+	+
Q8F613	Cytoplasmic membrane protein	LA_1499	4.214690149	4.395611249	+	+
Q8F237	Uncharacterized protein	LA_2940	4.214690149	4.633256821	+	+
Q8F7F0	Fibronectin type III domain-containing protein	LA_0995	4.214690149	4.702435914	+	+
G1UB25	Outer membrane lipoprotein LipL21	*lipL21*, LA_0011	3.859422	3.31019468	+	+
Q8F1S8	Hemolysin	LA_3050	3.048422	2.12035194	+	+
Q8F9H3	OmpA family lipoprotein	LA_0222	2.907422	1.985864701	+	+
G1UB30	OmpL1	LA_3138	2.903422	1.883620816	+	+
Q8EZX2	Fimh-like protein	LA_3729	2.8103932	1.727702673	+	+
Q8EZK5	Ig-like domain-containing protein	LA_3848	−1.728660207	−2.13289427	+	+
Q8F2C6	Flagellar hook protein FlgE	*flgE*, LA_2848	−2.59516801	−2.19759996	+	+
Q8F1M9	LenB	LA_3103	−3.852825595	−2.321928095	+	+
Q8EZE1	Putative lipoprotein	LA_3913	−4.5270496	−2.506352666	+	+
Q8F974	Fibronectin binding protein	LA_0322	−4.610809413	−2.506352666	+	+
Q8EZH2	Putative lipoprotein	LA_3881	−4.939228501	−2.910501849	+	+
Q8F8Q0	Uncharacterized protein	LA_0505	−7.074578	−3.878321443	+	+

## DISCUSSION

Understanding *Leptospira* pathogenesis is crucial for identifying the critical factors involved and devising better strategies to control infection. During infection in particular host, *Leptospira*, similar to other pathogens, also expresses multiple genes that are necessary for coordinated modulation of different phases of the disease, including adhesion, host penetration, and survival (29). The pathogen genes modulated during the interaction with the host could be critical virulence factors that might be involved at various stages of the progression of disease. Hence, identification and characterization of these factors will not only provide insight into pathogenesis but may also lead to the identification of targets for developing improved vaccines and diagnostics against this zoonosis. Previous attempts to identify such factors using omics relied only on transcriptomic or proteomic analyses of macrophages infected with *Leptospira*. Although these studies provided some insight into pathogenesis, poor correlation of transcriptome and proteome data failed to identify these critical factors (30).

In the present study, an integrated multi-omics approach was used to identify the candidate genes involved in host adhesion, virulence, and survival that are differentially regulated at both transcriptome and proteome levels upon interaction of *Leptospira* with human macrophages. RNA-Seq analysis revealed DEGs involved mainly in metabolic pathways (carbohydrate, energy, nucleotide, lipids, amino acids, vitamins, and secondary metabolites). Fifteen DEGs were related to the bacterial secretion system, fourteen to bacterial chemotaxis, and twelve genes each in the two-component system and flagellar assembly. Eight DEGs were known to be involved in quorum sensing (see Fig. S1A). Using gene ontology (GO) enrichment analyses, we found that the most significantly enriched GO biological terms belong to (i) the cellular process (SmpB, DnaK, RpmG, ProB, PrfB, etc.), (ii) chemotaxis (CheA, CheW, FliM, FliL, MotA, and MotB), (iii) localization (SecE, SecA, SecD, SecF, SecG, FliQ, FlgE, etc.), and (iv) locomotion, transport, and response to stimulus (MutS, UvrC, and UvrA) (see Fig. S1B). Previous studies evaluating the transcriptional response of Leptospira biflexa by RNA-Seq revealed genes regulating motility, sugar/lipid metabolism, iron scavenging, and outer membrane formation that were upregulated, while those of DNA replication and cell division were downregulated (31). Integrated analysis of genomics and RNA-Seq of Leptospira santarosai serovar Shermani- and Leptospira interrogans serovar Copenhageni Fiocruz (LIC)-infected HK-2 cells revealed high upregulation of many outer membrane lipoprotein genes, such as LipL32, LipL21, and LipL36, in different pathogenic and intermediate *Leptospira* spp., which is consistent with our findings (32).

Our proteomic analysis (see Fig. S2) shows modulation of several proteins involved in metabolic pathways, indicating the reprogramming of metabolic gene expression to adapt to host environmental conditions. Thus, the expression of LA_2309, which is fatty acid coenzyme A (CoA) ligase involved in fatty acid degradation, is an absolute requirement since Leptospira interrogans serovar Lai (LA) cannot utilize glucose or other sugars as carbon sources in the host environment and thus degrades long-chain fatty acids as an alternative (33, 34). Further, the downregulation of proteins involved in oxidative phosphorylation, such as cytochrome *caa*_3_ oxidase subunit II and cytochrome *c* oxidase polypeptide I, suggests that the bacteria reduce their requirement for oxygen during interaction with the host cell (35). However, several proteins of metabolic pathways, such as those involved in nucleotide metabolism (LA_ 3080 [adenylosuccinate lyase] and LA_3296 [uridylate kinase]) and tryptophan metabolism-like catalase (KatE), were upregulated. KatE plays a critical role in pathogenesis since it is involved in the degradation of H_2_O_2_, thus resisting oxidative killing of bacteria by phagocytes (36). In addition, *Leptospira* also drastically modulates the proteins involved in signal transduction, chemotaxis, flagellar assembly, secretion system, quorum sensing, etc. In contrast to other spirochetes, L. interrogans serovars Copenhageni Fiocruz and Lai have a two-component signal transduction system that includes histidine kinases and response regulators (35). Our proteomics data reveal the upregulation of eight proteins in a two-component system, which includes the ABC-type phosphate transport system protein and two methyl-accepting chemotaxis proteins (MCPs), except the chemotaxis-specific methyl transferase CheB, which was downregulated (see Fig. S3A). In most of bacterial species, chemotaxis is mostly conserved. MCPs play a major role in receiving and transmitting the chemotactic signal (37). In our data, 2 MCPs were upregulated. These MCPs might be involved in transmitting signals to other chemotaxis proteins such as CheA and CheW, leading to their activation by autophosphorylation and subsequent interaction with flagellar motor switch complex to alter the motility apparatus or rotating direction (38–41). Phosphorylated CheA also phosphorylates another chemotaxis-specific methyltransferase, CheB, which is downregulated in our data and plays an important role in chemotaxis memory or adaptation by demethylation of MCPs to adjust to a new signal (42–44). Further, proteins involved in flagellar assembly and motility, including the endoflagellar motor switch protein FliN, the endoflagellar biosynthesis pathway protein FliQ, and the flagellar hook protein FlgE, were downregulated, suggesting reduced motility of bacteria during the interaction with macrophages (30, 45) (see Fig. S3B). Secretion systems have not been well characterized or experimentally validated in *Leptospira* (46). Based on some *in silico* analysis, it has been shown that L. interrogans serovars Lai and Copenhageni possess relatively complete type I and type II secretion systems and incomplete type III, IV, V, and VI secretion systems (33, 47, 48). Our proteomics data show the upregulation of TolC and TolC family proteins, which are involved in creating transperiplasmic channels of the type I secretion system and also the upregulation of hemolysin (LA_3050), which is involved in pulmonary hemorrhage and is exported by the type I secretion system (49–52). Pathogenic L. interrogans serovar Lai has nine Gsp (53) proteins that are an integral part of the type II secretion system and play a major role in secreting toxins and cell wall-degrading enzymes. Our proteomics data show that of nine Gsp proteins, seven (GspD, GspC, GspF, GspG, GspJ, GspK, and GspE) were upregulated during the interaction with the host cells (see Fig. S3C). GspF is the inner membrane component of the type II secretion system involved in interaction with a cytoplasmic hexameric ATPase (GspE) of the type II secretion system (54). Sec translocase plays a major role in the export of proteins with N-terminal signal peptides, including lipoproteins across the inner membrane, and our data show the upregulation of orthologs of all the necessary factors of this complex (see Fig. S3D), suggesting that bacteria might activate and secrete proteins through the Sec translocase system during interaction with macrophages (55).

Transcriptomic and proteomic analyses, when performed alone, have several limitations in studying host-pathogen interaction, since the mRNA level does not always correlate with protein expression (56, 57). For instance, LipL36 protein level is reduced under iron depletion conditions, but not *lipL36* transcription (30). Therefore, integrating transcriptome and proteome analyses can provide robust and reproducible data to develop system-level understanding. In our study, we integrated both data set and conjoint analyses to reveal 677 common transcripts and protein pairs (Fig. 4A) in which 87 pairs were upregulated and 43 pairs were downregulated, whereas 23 common pairs were upregulated in the transcriptome but downregulated in the proteome (Fig. 4B and C). It is not very straightforward to speculate the biological causes of these results, since these might be associated with posttranscriptional regulation or posttranslational modifications and/or protein stability. Although no genes were downregulated in the transcriptome and upregulated in the proteome (Fig. 4B), a discrepancy in the number of identified genes or proteins and their expression levels was found that has significant implications. Protein is the final downstream product of multiple regulated interactions between genes and transcripts. However, it may not be necessary to obtain protein for all the identified transcripts, and protein levels may also not necessarily correspond to similar transcript levels. Considering that the mechanism underlying the pathogenesis is complex and involves several processes, including adhesion, invasion, secretion, and virulence, we further divided 130 common transcripts and proteins into four categories (adhesin, pathogenic, invasion, and secretory) using *in silico*-based analysis (Table 1). Since recognition and binding to host surface components is a crucial step in pathogenesis, we chose outer membrane/surface proteins in conjoint analysis since they are most likely interacting with the host surface molecules and help in the entry and dissemination of bacteria. Of 21 proteins that were predicted to be adhesins, 17 are localized to periplasmic, outer membrane, and extracellular locations (see Fig. S4A and B). Of these, nine were upregulated, and eight were downregulated. Several well-known and characterized protein/virulence factor/adhesins were significantly upregulated in our study, including LigB, Loa22, OmpL1, LipL21, hemolysins, fibronectin type III domain-containing protein (LA_0995), cytoplasmic membrane protein (LA_1499), and FimH-like protein (LA_3729). LigB is an adhesin known to interact with various components of extracellular matrix (ECM); however, the LigB mutant is not attenuated in virulence (34, 58–61). The cytoplasmic membrane protein (LA_1499) contains a bacterial immunoglobulin-like domain 5 and might be involved in a nonclassical secretion pathway as predicted by *in silico* analysis (53, 62). LipL21 is one of the abundant outer membrane lipoproteins in pathogenic L. interrogans serovar Copenhageni (63), which is highly expressed during infection and binds to Toll-like receptor 4 (TLR4) and complement regulators for modulating the innate response (9, 64, 65). Loa22 contains an OmpA domain and has been shown to bind to ECM components such as fibronectin and collagen and also TLR2 to induce proinflammatory response (66, 67). Loa22 is expressed during infection and its mutant was attenuated in virulence in hamsters and guinea pigs (66, 68, 69). FimH-like protein (LA_3729) is type I bacterial adhesin containing immunoprotective domain DUF1566 and binds to mannosylated glycoproteins on the surfaces of human and murine bladder cells to facilitate bacterial colonization, invasion, and the formation of biofilm (70–73). OmpL1 is an ECM binding protein known to be present in all pathogenic Leptospira kirschneri strain RM52 species is involved in cross-protective immunity (74–79). Our conjoint analysis revealed downregulation of several proteins such as LA_3848, FlgE, LenB, two putative lipoproteins (LA_3913 and LA_3881), fibronectin-binding protein (LA_0322), and two uncharacterized proteins (LA_0505 and LA_0100) that are extracellular, outer membrane, and periplasmic and are predicted to have adhesion properties. Our result suggests that the LEPIRGA interaction with the macrophages was a critical factor that contributes to the differential regulation of these OMPs, which are predicted to have adhesin properties since the expression of these proteins did not change in LEPIRGA strains incubated in medium alone. Since most of the proteins identified in our study are known to be involved in the activation of macrophages or evasion from complement system, modulation of their expression indicates an immune evasion strategy adapted by pathogenic *L. interrogans* serovar Icterohaemorrhagiae (65, 80).

Invasins play a crucial role in mediating entry of the bacteria in the host cell and in our analysis, we found 6 proteins (collagenase, Mce, hemolysin, putative phospholipase, Sph1, and Sph2), which are upregulated and are predicted to be invasins or having invasive ability (27, 81) (Table 1). Collagenase plays important role in the invasive ability of many pathogenic bacteria (82–84). Pathogenic L. interrogans serovar Lai also produces a collagenase (ColA) with hydrolytic activity whose expression and secretion levels are significantly increased when coincubated with human umbilical vein endothelial cells (HUVECs) and renal epithelial cells (HEK293). The *colA* gene knockout mutant showed a low transcytosis through HUVEC and HEK293 monolayers and decreased leptospires in blood, lung, liver, and kidney in hamsters compared to the wild-type strain (85). Mce (mammalian cell entry) proteins previously reported in Mycobacterium tuberculosis and having role in invasion have also been reported to be present in pathogenic L. interrogans serovar Hardjo subtype Hardjoprajitno (LIHH) (33, 86). Mce binds to the α5β1 and αVβ3 integrins and mediates the *Leptospira* internalization into macrophages (81). Pathogenic *Leptospira* (LIC, LA, and LIHH) genome consists of five sphingomyelinase genes (*sph-1* to *sph-4*, as well as *sphH*), out of which *sph-2* is reported to be most secreted during infection and is involved in mediating the invasion of bacteria in lung and liver cells through clathrin-mediated endocytosis, thereby inducing apoptosis (33, 35, 87, 88). Next, in our conjoint analysis, we found that 19 proteins predicted to be secretory are upregulated (Table 1; see Fig. S4B); these include lipoproteins (Lipl32, Lipl21, and Lipl45), hemolysins (LA_3050, Sph1 and Sph2) and outer membrane (OmpL1, Loa22, LigB, and LA_3729) proteins, which are potential virulence factors and interact with the host proteins (50).

To confirm the expression of RNA-protein pairs in integrated analysis, we chose eight genes to validate by using qRT-PCR. Despite the recognized expression difference, the concordance of omics data for identified genes is still worthwhile, and the qRT-PCR results for candidate genes support the omics data very well (Fig. 6; see also Fig. S5).

In conclusion, by studying LEPIRGA macrophage interaction using an integrated multi-omics approach, we identified several proteins with virulence and host adhesive properties that are modulated, indicating their involvement in first-line host adhesion and interaction. The integrated analysis not only identified proteins that could be critical factors involved in pathogenesis but also provided new insights in understanding *Leptospira*-host interaction, which might have not been possible using analysis at either the mRNA or the protein level alone. However, validation of these critical factors is important to define their precise role in *Leptospira* pathogenesis.

## MATERIALS AND METHODS

### *Leptospira* and THP-1 cell culture and infection experiment.

L. interrogans serovar Icterohaemorrhagiae strain RGA (LEPIRGA) was cultured in EMJH medium (BD) and grown to mid-log phase (~10^8^ *Leptospira* organisms/mL) aerobically at 28°C without agitation. The human monocytic cell line (THP-1), collected from the American Type Culture Collection (ATCC; Rockville, MD) was cultured using RPMI 1640 medium (Gibco Laboratories, Grand Island, NY) containing 10% heat-inactivated fetal bovine serum (FBS; Gibco), 100 U/mL penicillin and 100 mg/mL streptomycin (Sigma Chemical Co., St. Louis, MO) at 37°C in an atmosphere of 5% CO_2_ until reaching 70 to 80% confluence. The cells were then treated with phorbol-12-myristate-13-acetate (PMA; Sigma) at 10 ng/mL for 48 h to induce differentiation into macrophages. The cells were then washed with 1× phosphate-buffered saline (PBS) and grown in antibiotic-free RPMI medium for additional 2 days. The LEPIRGA culture was pelleted down and washed twice with 1× PBS and then resuspended in antibiotic-free RPMI medium for 12 h before infection. LEPIRGA infection was given at a multiplicity of infection (MOI) of 1:100 for 24 h at 37°C and 5% CO_2_. LEPIRGA incubated in an antibiotic-free RPMI medium without cells at the same conditions was considered the control sample. Experimental sampling was done in triplicates for transcriptome and proteome processing.

### Blood collection.

The whole blood from random healthy individuals were collected with informed consent by staff of Gandhi Medical College, Hyderabad, Telangana, India, following Institutional Ethics Committee approved protocol (IEC/GMC/2021/03/25).

### Exposure of LEPIRGA to whole human blood.

We treated whole human blood (WHB) samples (*n* = 3) with LEPIRGA as described above and incubated them for 24 h at 37°C with 5% CO_2_. After 24 h of infection, the whole sample was transferred into tube containing equal volume of Histopaque 1077 (Sigma-Aldrich) and centrifuged at 400 × *g* for 30 min to separate the red blood cells. The plasma and buffy coat layer were transferred into a fresh 15-mL tube and centrifuged at 1,500 rpm for 10 min at room temperature to separate host cells from bacterial cells. The supernatant was transferred into a fresh 15-mL tube and centrifuged at 9,000 rpm for 10 min at room temperature to collect the live bacteria.

### Isolation of human blood primary macrophages.

Peripheral blood mononuclear cells (PBMCs) were isolated from whole blood by density gradient centrifugation using Histopaque 1077 (Sigma-Aldrich). PBMCs were cultured in complete RPMI medium for 24 h 5% CO_2_ incubator at 37°C to allow the monocytes to adhere to dish. Monocytes were differentiated into macrophages using 50 ng/mL M-CSF (Peprotech) in RPMI 1640 medium supplemented with 10% FBS, 100 U/mL penicillin, and 100 μg/mL streptomycin for 5 days at 37°C and 5% CO_2_. After differentiation, the macrophages were harvested by trypsinization, seeded into multiwell plates, and then infected with LEPIRGA as described above.

### RNA isolation, library preparation, and sequencing.

Total RNA was extracted from LEPIRGA in RPMI medium (control), and LEPIRGA-infected THP-1 cells at an MOI of 1:100 (test) at 24 h postinfection by using an RNeasy kit (Qiagen, Valencia, CA) according to the manufacturer’s protocol. Three replicates were prepared for both control and test samples. The integrity of isolated RNAs was assessed using an RNA NanoChip bioanalyzer (Agilent Technologies, Wilmington, DE) to ensure an RNA integrity value of ≥8. cDNA libraries were prepared using NEB Next Ultra-II Directional RNA Library Prep kit for Illumina (NEB) according to the manufacturer’s protocol. Illumina RNA-Seq libraries were prepared with a TruSeq RNA sample prep kit (Illumina, San Diego, CA) according to the manufacturer’s protocol. Paired-end sequencing was performed using an Illumina HiSeq 2500 instrument with a 2 × 150-bp setup.

### Protein extraction and sample preparation for LC-MS/MS.

Both control and test samples were lysed in AMBIC lysis buffer (50 mM ammonium bicarbonate with 0.1% sodium deoxycholate; Sigma) and then boiled at 100°C for 5 min, followed by cooling on ice for 5 min on ice before sonication (25 amplitude for 5 min with a 10-s on/off pulse) and centrifugation (14,000 rpm for 10 min at 4°C). The collected protein supernatant concentration was quantified using a BCA protein assay kit (Pierce), and 100 μg of protein was reduced with 10 mM dithiothreitol (Sigma) at 50°C for 45 min, followed by alkylation with 50 mM iodoacetamide (SRL) at room temperature for 20 min in the dark. Later, the samples were digested overnight with trypsin/LysC (Promega) at 37°C and acidified using formic acid (Pierce) for detergent removal, followed by centrifugation at 14,000 rpm for 10 min. The clear supernatants containing digested peptides were passed through a 10-kDa cutoff filter (Thermo Scientific) and vacuum dried using a SpeedVac. The obtained vacuum-dried peptides were resuspended into 20 μL of 5% acetonitrile (Pierce) with 0.1% trifluoroacetic acid (Pierce), followed by centrifugation for 10 min at the highest speed. The clarified vacuum-dried peptides were purified via Thermo Scientific Pierce C_18_ tip ready-to-use pipette-tip columns of C_18_ resin according to the manufacturer’s instructions. The eluate fractions were vacuum dried in the SpeedVac and stored at −20°C prior to liquid chromatography-tandem mass spectrometry (LC-MS/MS) data generation. All the samples were processed using the same experimental procedure.

### RNA-Seq data analysis.

The quality of generated RNA-Seq data were evaluated using FastQC v0.11.5 (https://www.bioinformatics.babraham.ac.uk/projects/fastqc/) and MultiQC v1.8 (89). Quality control was performed using Fastp v0.20.1 (90) software with the following parameters: –qualified_quality_phred = 30 –length_required = 70 –detect_adapter_for_pe. Only high-quality reads and sequences of more than 30 nucleotides were considered for further analysis. Bowtie v2.4.5 (91), with default parameters, was used for alignment on the reference genome of L. interrogans serovar Lai 56601 (LEPIN). Due to the lack of a reference genome for LEPIRGA, we used LEPIN from the MicroScope Platform (https://mage.genoscope.cns.fr/microscope/home/index.php). FeatureCount (92) was used for the abundance estimation from the Subreads package (parameters: -t gene -g locus_tag -s 1). Then, differentially expressed transcripts were identified between control and test samples using DESeq2 (93). Differentially expressed transcripts were identified at a *P*_adj_ of ≤0.05 and a Log_2_FC of ≥+1 and ≤–1. The analyses were carried out at the National Institute of Animal Biotechnology, Hyderabad, India.

### LC-MS/MS data generation and data analysis.

Peptides were resuspended in 0.1% formic acid, and 1 μg of the peptide was subjected to LC-MS/MS on an Ultimate 3000 RSLC nano-liquid chromatography system (Dionex) coupled to a QExactive HF mass spectrometer (Thermo Fisher Scientific, Inc.). The samples were separated on PepMap RSLC (C_18_, 2 μm, 100 Å, 75-μm inner diameter × 50-cm column) at a flow rate of 0.3 μL/min. Peptides were eluted by a linear gradient from 5 to 95% acetonitrile over 80 min and directly sprayed into a QExactive HF mass spectrometer equipped with a nanoFlex ion source (Thermo Fisher Scientific) at a spray voltage of 2 kV. Full-scan MS spectra (375 to 1,500 *m/z*) were obtained at a resolution of 60,000 at an *m/z* ratio of 200, a maximum injection time of 60 ms, and an AGC target value of 3 × 10^6^. Up to 20 of the most intense peptides per full scan were isolated using a 1.2-*m/z* isolation window and fragmented using higher-energy collisional dissociation (normalized collision energy of 27). MS/MS spectra were obtained with a resolution of 15,000 at *m/z* 100, a maximum injection time of 100 ms, and an AGC target value of 10^5^. Ions with charge states of 1 and >6 and ions with unassigned charge states were not considered for fragmentation. Dynamic exclusion was set to 20 s to minimize repeated sequencing of already-acquired precursors. Raw files were analyzed using MaxQuant (v2.1.3.0) using label-free quantification, and spectra were selected using default parameters. Database searches were performed against a trypsin-digested LEPIN protein database (due to the lack of a protein database information for LEPIRGA in UniProt, we used LEPIN as a reference proteome) and FASTA file of common contaminants (contaminants.fasta) was provided for quality control.

### Bioinformatics analysis.

DEPs were further annotated through gene ontology, such as biological processes, molecular activity, and cellular components, by using the PANTHER gene classification system (Fisher exact test; false discovery rate [FDR] ≤ 0.05) (94). Pathway analysis was done using KEGG (95); a hypergeometric test determined an FDR of ≤0.05 using hypeR (96). Protein abundances were visualized as a heatmap, generated by the ClustVis webtool (97) using hierarchical clustering with Euclidean distance metrics and complete linkage. The CELLO v2.5 tool was used to identify the subcellular localization of proteins (98). To predict the adhesin nature of proteins, the Dynamic Vaxign Analysis *In Silico* Tool (https://violinet.org/vaxign/; [99]) was used. To predict pathogenic proteins, MP3 web software (http://metagenomics.iiserb.ac.in/mp3/) was used (100). Mapping of protein-protein interaction to know the functional protein association networks was conducted using the STRING database v11.5 (101). The evidence mode was set at 0.7, a high confidence level, and all the search parameters were included, such as text mining, experiments, databases, coexpression, neighborhood, gene fusion, and co-occurrence. Generated networks were imported into Cytoscape v3.8.2 (102) for further analysis.

### qRT-PCR validation of candidate genes.

Eight RNA-protein pairs from integrated analysis that were highly modulated were selected for validation of expression by qRT-PCR using specific primers (see Table S7 in the supplemental material). THP-1 macrophages/HBPMs/HWB were infected with LEPIRGA and at 2, 6, 12, and 24 h postinfection, and RNA was isolated from the samples. cDNA was synthesized using a PrimeScript first strand cDNA synthesis kit (TaKaRa) according to the manufacturer’s instructions. qRT-PCR was performed in 96-well microtiter plates on the Bio-Rad CFX96 Touch real-time PCR detection system. The two-step amplification was performed in a 10-μL reaction volume containing 50 ng of cDNA, 10 μM concentrations of each primer, and 5 μL of iTaq Universal SYBR green Supermix (Bio-Rad). Samples were run in triplicate, and data were analyzed using a sequence detection system (Bio-Rad). The experimental data are presented as the fold changes in gene expression in test relative to control samples at various time points. mRNA levels of the analyzed genes were normalized to 16S RNA present in each sample. The absence of contamination was confirmed by running no-template and no-RT controls. All qRT-PCR analyses were performed in triplicates, and a pairwise Δ*C_T_* method algorithm was used to evaluate gene expression stability.

### Data availability.

Data that support the findings of this study are available from the corresponding authors upon reasonable request. All the RNA-Seq data are publicly available through NCBI SRA repositories (PRJNA755864). Proteomics Raw files (LC-MS/MS-proteomics-*Leptospira* pathogenesis-human macrophages) and statistical analysis data are publicly and freely accessible from the Zenodo repository (https://doi.org/10.5281/zenodo.7340320).
