# Supplementary material for: Use of an Integrated Multi-Omics Approach To Identify Molecular Mechanisms and Critical Factors Involved in the Pathogenesis of Leptospira
Source: Microbiol Spectr. 2023 Feb 28;11(2):e03135-22. doi: 10.1128/spectrum.03135-22 (PMC10100824; doi:10.1128/spectrum.03135-22)
Supplement: Supplemental file 1 — Fig. S1 to S5 and Tables S1, S3, and S5 to S9. Download spectrum.03135-22-s0001.pdf, PDF file, 1.5 MB [file spectrum.03135-22-s0001.pdf]

Supplementary Information (Data)

Integrated multi-omics approach to identify molecular mechanism and critical factors involved in pathogenesis of *Leptospira*

Kavela Sridhar [1¶], Pallavi Vyas[1,2¶], Jusail C.P.[1,2], Sandeep K. Kushwaha[3], Subeer S. Majumdar[4*], Syed M. Faisal[1,2*]

*Corresponding authors: Syed M. Faisal (faisal@niab.org.in) and Subeer S. Majumdar (subeer@niab.org.in), National Institute of Animal Biotechnology, Hyderabad-500032, India.

Supplementary Figure Legends:

Supp. Fig1: Gene ontology and KEGG pathway analysis of transcriptomics DEGs. A) The functional classification of the 314 DEGs was analyzed by GO enrichment analysis. B) KEGG pathway analysis was performed to investigate the significant pathways enriched by the 314 differentially expressed genes in transcriptomics data.

Supp. FigS2: Gene ontology and KEGG pathway analysis of proteomics DEPs. A) The functional classification of the 244 DEPs was analyzed by GO enrichment analysis. B) KEGG pathway analysis was performed to investigate the significant pathways enriched by the 244 differentially expressed proteins in proteomics data.

Supp. FigS3: Enriched KEGG pathway in Proteomics. Enriched KEGG pathway showing up and downregulated genes. A) Bacterial Chemotaxis. B) Flagellar Assembly. C) Protein Export. D) Bacterial Secretion System.

Supp. FigS4: Conjoint Analysis of transcriptome and proteome. A) Subcellular localization of 130 common RNA-Protein pair. B) Insilico prediction (Pathogenic, Secretory, Invasin, Adhesin) of RNA-Protein Pairs. C) Protein-Protein Interaction (PPI) network using a STRING database and network analysis

Supp. FigS5: RT-qPCR Validation. A) Graphical representation of 08 RNA-Protein pairs of LEPIRGA at different times points after interaction with the Host (THP1/HBPM/HWB).

Supp. Fig. S1

A. KEGG Pathway

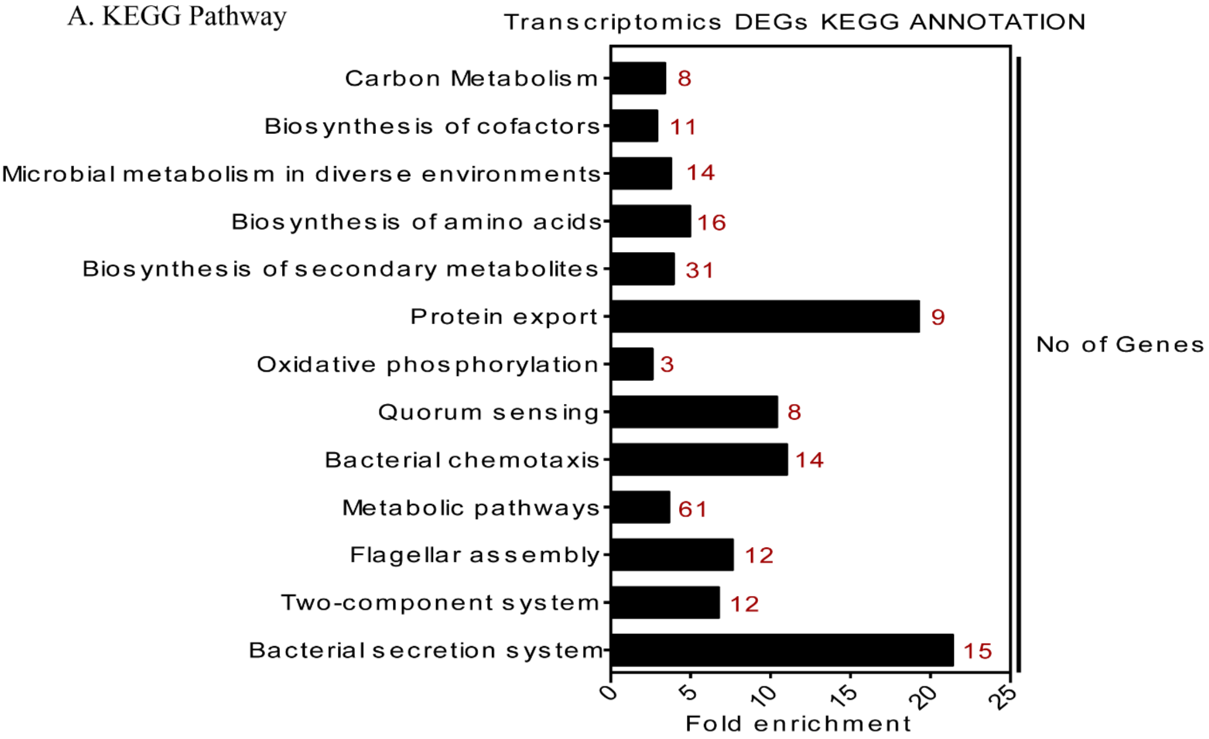

B. Gene Ontology

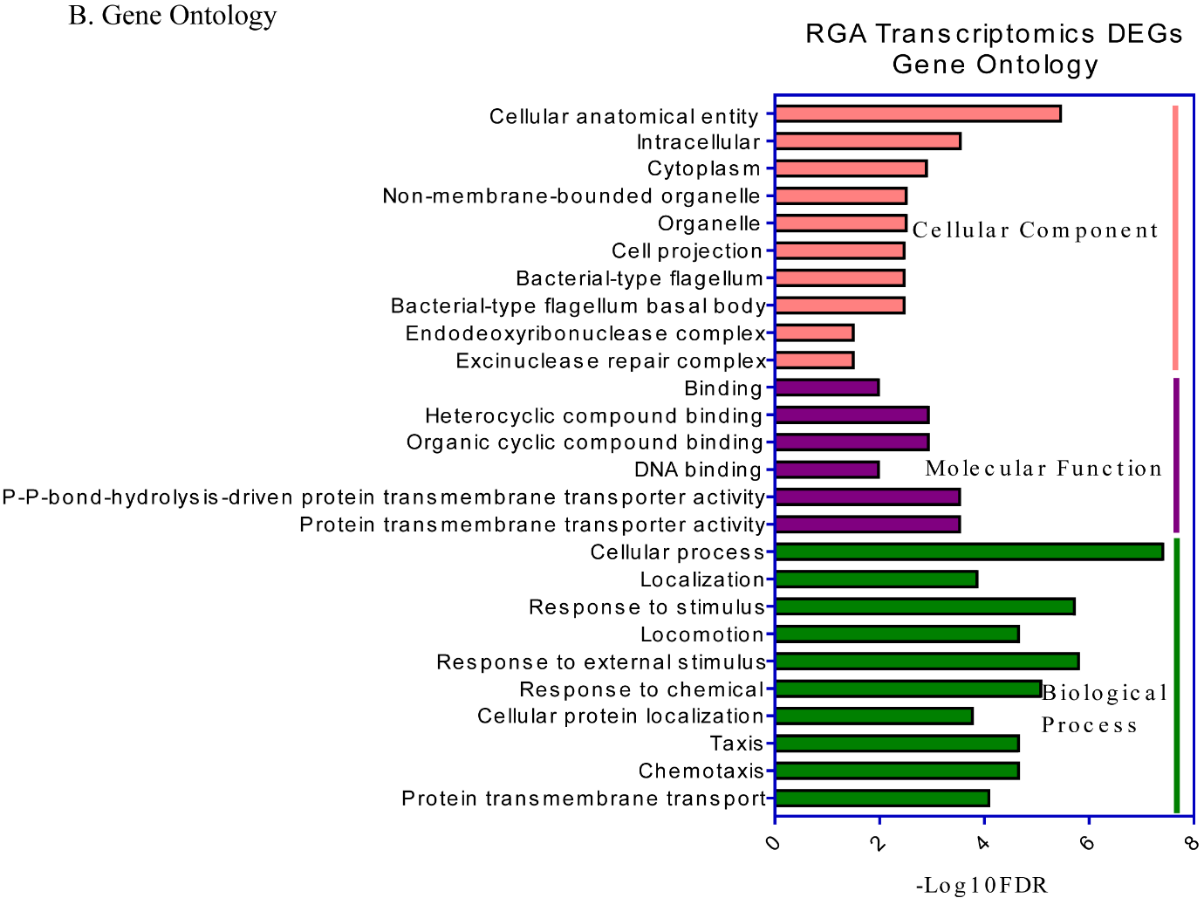

Supp. Fig. S2

A. Gene Ontology

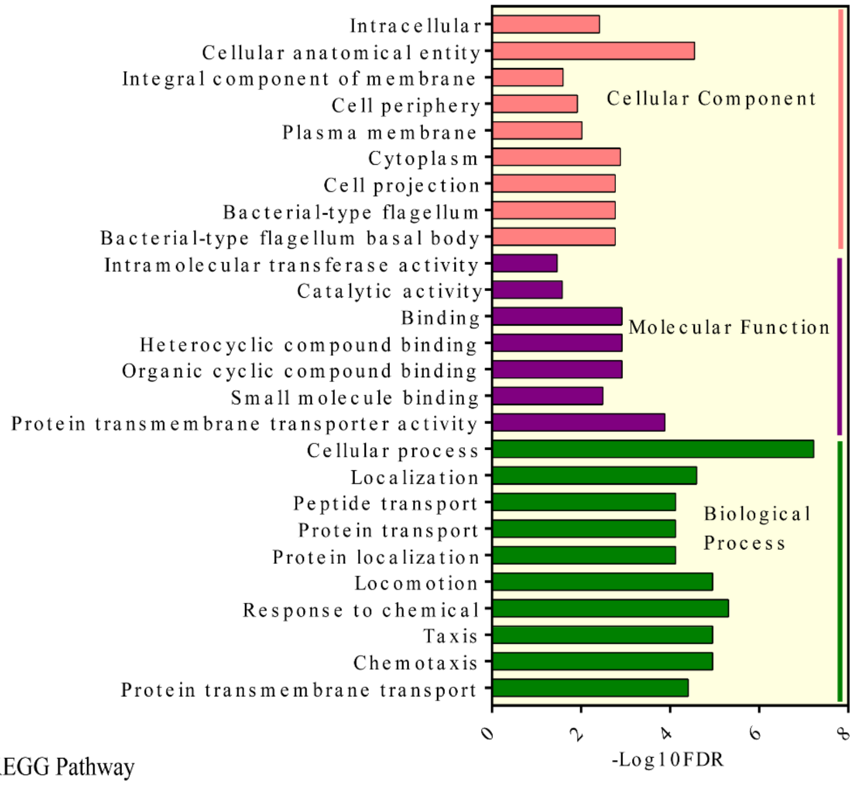

B. KEGG Pathway

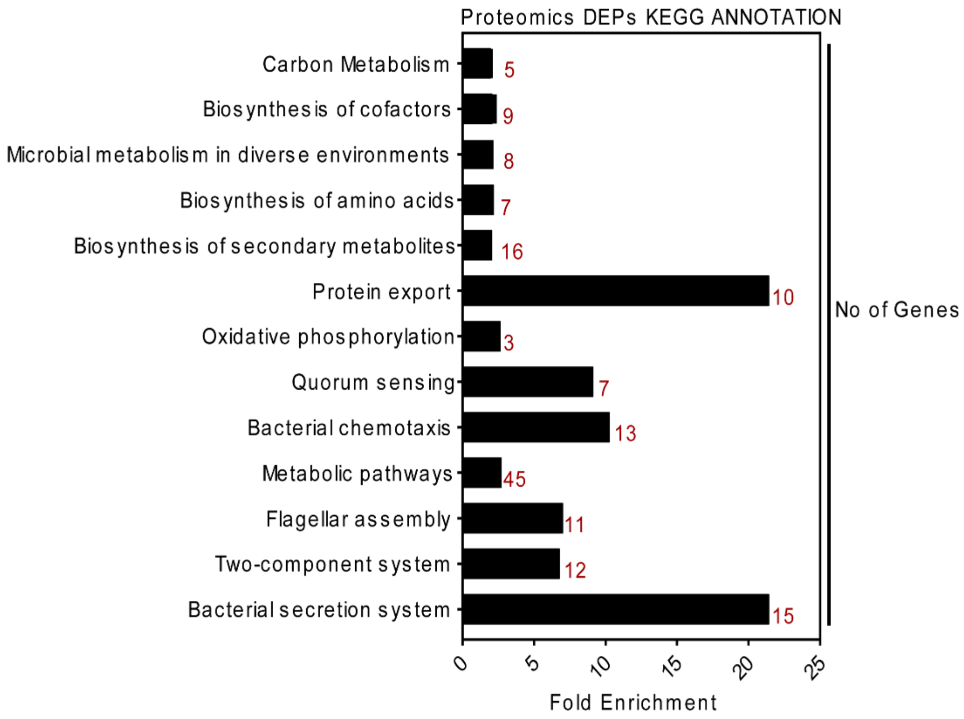

Supp. Fig. S3

A) Bacterial Chemotaxis

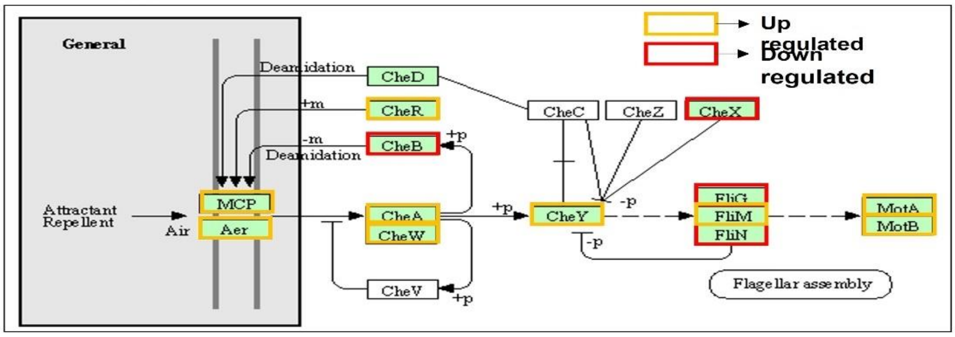

B) Flagellar Assembly

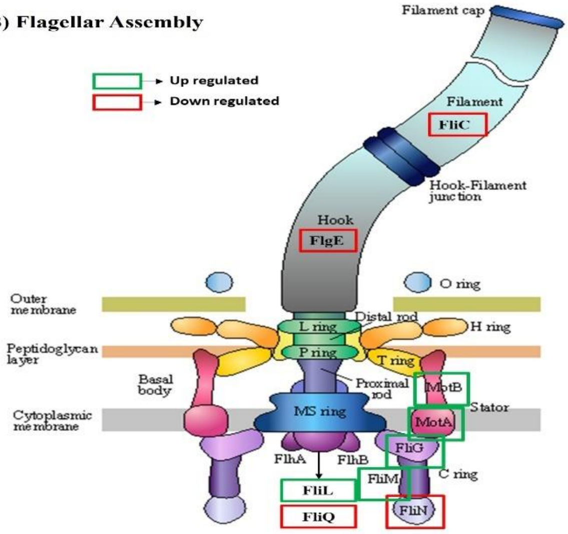

D) Protein Export

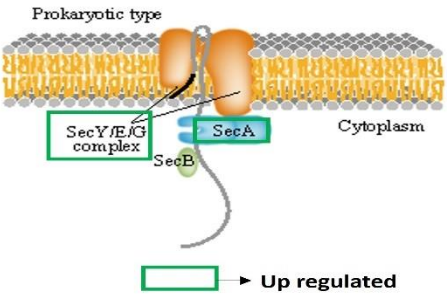

C) Bacterial Secretion System

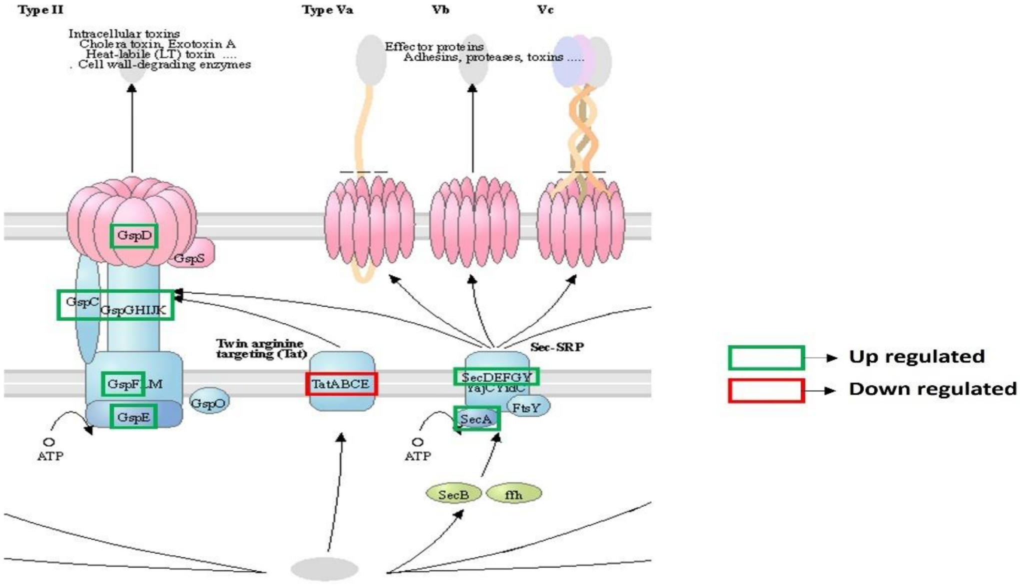

Supp. Fig. S4

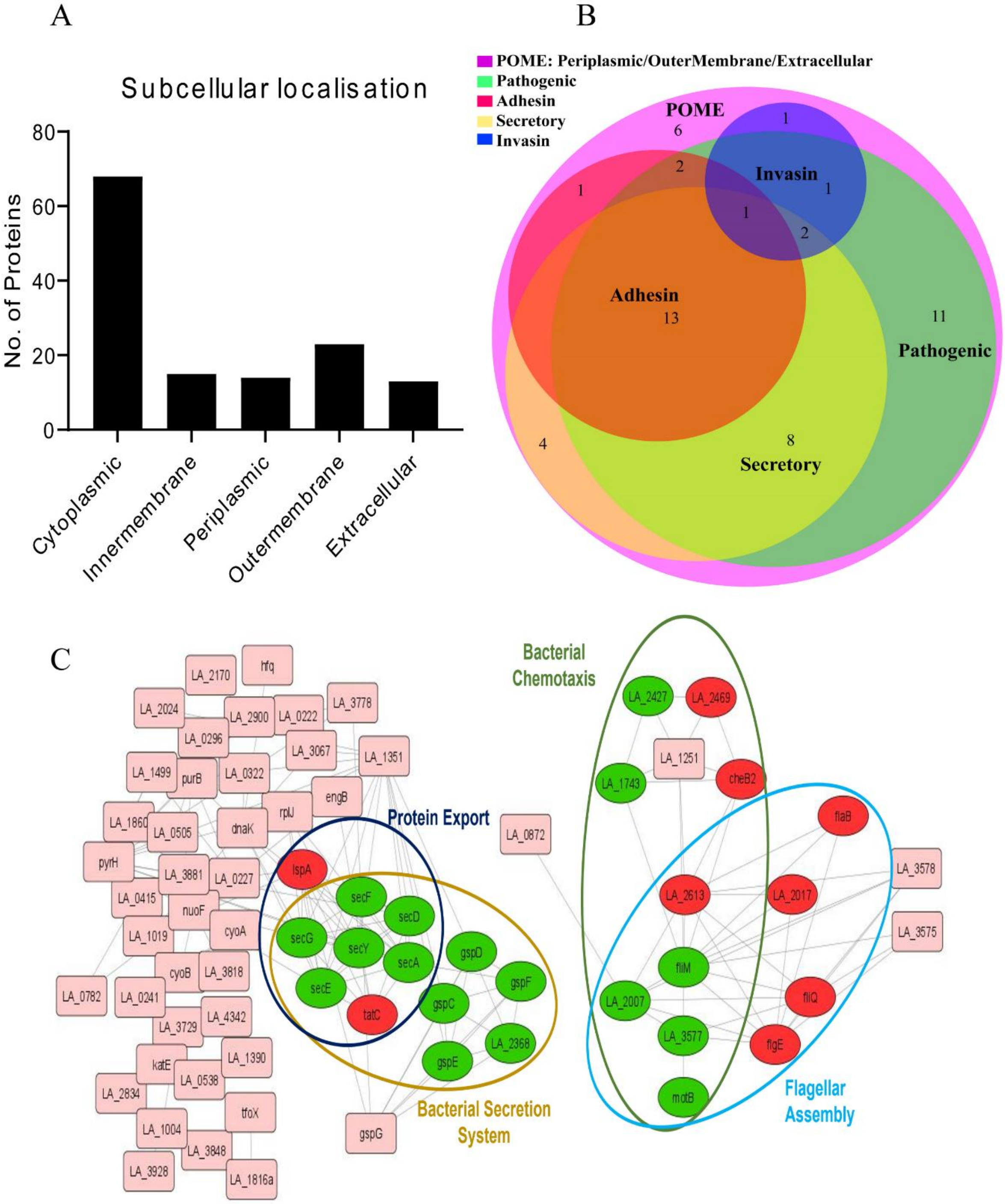

Green - Upregulated , Red - Downregulated

number of nodes: 130
number of edges: 162
average node degree: 2.49
avg. local clustering coefficient: 0.361
expected number of edges: 88
PPI enrichment p-value: 1.21e-12

Supp. Fig. S5

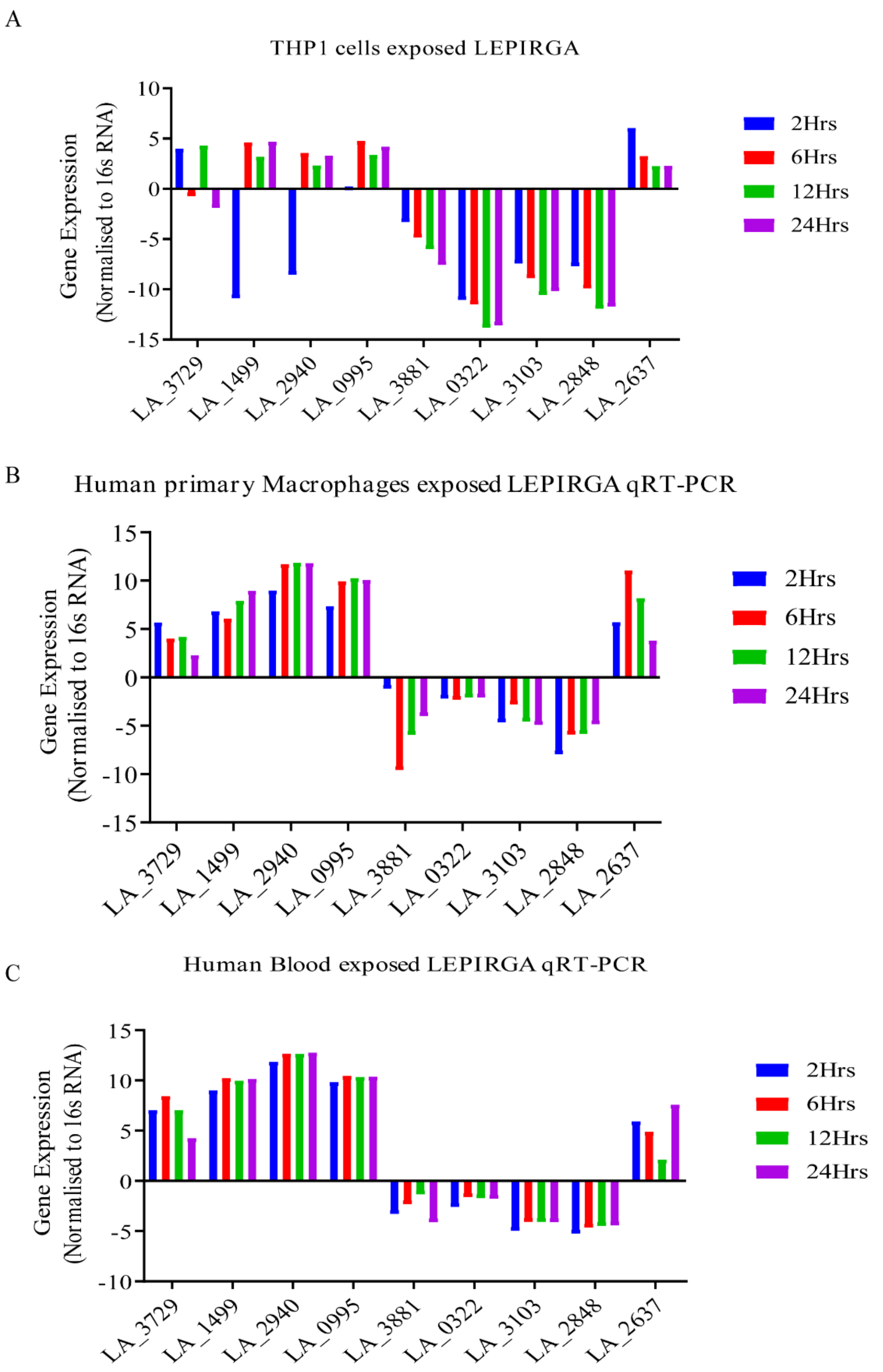
A
THP1 cells exposed LEPIRGA
Gene Expression
(Normalised to 16s RNA)
10
5
0
-5
-10
-15
LA_3729
LA_1499
LA_2940
LA_0995
LA_3881
LA_0322
LA_3103
LA_2848
LA_2637
2Hrs
6Hrs
12Hrs
24Hrs
B
Human primary Macrophages exposed LEPIRGA qRT-PCR
Gene Expression
(Normalised to 16s RNA)
15
10
5
0
-5
-10
-15
LA_3729
LA_1499
LA_2940
LA_0995
LA_3881
LA_0322
LA_3103
LA_2848
LA_2637
2Hrs
6Hrs
12Hrs
24Hrs
C
Human Blood exposed LEPIRGA qRT-PCR
Gene Expression
(Normalised to 16s RNA)
15
10
5
0
-5
-10
LA_3729
LA_1499
LA_2940
LA_0995
LA_3881
LA_0322
LA_3103
LA_2848
LA_2637
2Hrs
6Hrs
12Hrs
24Hrs

Supplementary Table 1: (Quality assessment and quality control)

Sample Name	Raw				Clean			
	Nucleotide number	Sequences flagged as poor quality	Read length	GC %	Nucleotide number	Sequences flagged as poor quality	Read length	GC %
RGAa37-T1_1	14026286	0	151	40	12851676	0	31-151	37
RGAa37-T1_2	14026286	0	151	40	12851676	0	31-151	37
RGAa37-T2_1	22291882	0	151	39	18822454	0	31-151	37
RGAa37-T2_2	22291882	0	151	39	18822454	0	31-151	37
RGAa37-T3_1	28588731	0	151	39	25390303	0	31-151	37
RGAa37-T3_2	28588731	0	151	39	25390303	0	31-151	37
THPI_RGA-T1_1	19534902	0	151	41	10529532	0	31-151	37
THPI_RGA-T1_2	19534902	0	151	41	10529532	0	31-151	37
THPI_RGA-T2_1	23926187	0	151	41	12680764	0	31-151	37
THPI_RGA-T2_2	23926187	0	151	41	12680764	0	31-151	37
THPI_RGA-T3_1	19206835	0	151	41	10004784	0	31-151	37

THPI_RGA-T3_2	19206835	0	151	41	10004784	0	31-151	37

Supplementary Table 2: (Transcriptomics supplementary file.xlsx)

As separate attachment

Supplementary Table 3: Top 50 Differentially expressed Leptospira genes upon Host Interaction

UniRef100	Protein ID	Protein names	Gene names	Log2FC	Log 10 P Value
UniRef100_Q8F349	Q8F349	Glycosylhydrolase	bglX	11.93542	5.591149265
UniRef100_Q72MQ7	Q8EZC7	Acyl-CoA dehydrogenase	caiA	8.362422	2.351635029
UniRef100_Q8F6Q0	Q8F6Q0	Chemotaxis protein CheA (EC 2.7.13.3)	cheA	7.645422	3.386191757
UniRef100_A0A0F6HG36	Q8F5D9	Methylase of chemotaxis methyl-accepting protein	cheR	7.025422	1.940877138
UniRef100_A0A1X8WJY4	Q8F3H6	Chemotaxis protein CheW	cheW	7.006422	5.223947897
UniRef100_A0A0E2CZ65	Q8F4N3	Flagellar motor switch protein FliG	fliG	5.215422	2.247142211
UniRef100_M6RS94	Q8F0C1	Flagellar protein FliL	fliL	4.765422	2.602991392
UniRef100_A0A8G0YXY6	Q8F8F9	Transcriptional regulator	LA_0598	4.21469	14.68588844
UniRef100_Q8F927	Q8F927	Uncharacterized protein	LA_0370	4.21469	3.856866429
UniRef100_A0A1X8WI59	Q8F7R5	Uncharacterized protein	LA_0879	4.21469	3.382939793
UniRef100_Q8EZS3	Q8EZS3	LigB-like protein	LA_3778	4.21469	2.930786396
UniRef100_Q8F237	Q8F237	Uncharacterized protein	LA_2940	4.21469	2.86966727
UniRef100_A0A0C5XH69	Q8F8R1	Putative lipoprotein	LA_0494	4.21469	2.733925425
UniRef100_Q8F613	Q8F613	Cytoplasmic membrane protein	LA_1499	4.21469	2.67269605
UniRef100_A0A0E2DJC8	Q8F7F0	Fibronectin type-III domain-containing protein	LA_0995	4.21469	1.971691521
UniRef100_A0A868AUI0	Q8F4G0	Flagellar motor switch protein FliM	fliM	4.204422	1.420038219

UniRef100_A0A868AW11	Q8F3M5	Type II secretory pathway component protein C	gspC	4.151422	3.411403566
UniRef100_A0A8G0YXA1	Q8F3M6	Type II secretory pathway component protein D	gspD	4.128422	6.264589298
UniRef100_Q8EXG7	Q8EXG7	Na+/solute symporter	putP	4.112414	1.322130638
UniRef100_Q8F3B8	Q8F3B8	Predicted membrane protein involved in D-alaninealginate export/acetyltransferase of MBOAT family	dltB	4.096942	10.1027082
UniRef100_A0A1X8WNH3	Q8F3M7	Protein-secreting ATPase (EC 7.4.2.8)	gspE	4.090422	2.429927169
UniRef100_A0A868AW95	Q8F3M8	Type II secretory pathway component protein F	gspF	4.052422	7.561724945
UniRef100_A0A8G1DY50	Q8F3M9	Type II secretory pathway component protein G	gspG	4.039422	1.616492211
UniRef100_Q72Q67	Q8F5Z7	RNA-binding protein Hfq	hfq	4.036422	2.513584372
UniRef100_A0A0E2D7B3	Q8F808	TM2 domain-containing protein	LA_0782	3.928502	6.712955718
UniRef100_Q8F3D6	Q8F3D6	Putative lipoprotein	LA_2470	3.928502	6.094044473
UniRef100_A0A1B9FGS8	Q8F528	Catalase (EC 1.11.1.6)	katE	3.921422	2.316355223
UniRef100_Q4PLB0	G1UB25	Outer membrane lipoprotein LipL21	lipL21	3.859422	1.877842055
UniRef100_Q6J0P4	O34094	LipL32	lipL32	3.769422	4.385869671
UniRef100_M3HDK9	Q8F3V5	LipL45	lipL45	3.712422	2.103402618
UniRef100_A0A1B9FPV7	Q8F0B9	Endoflagellar motor protein A	motA	3.694422	7.066798697
UniRef100_A0A8G0Z2K8	Q8F6B8	Uncharacterized protein	LA_1390	3.681176	2.024490522
UniRef100_A0A0E2DGK7	Q8F3Z3	Predicted membrane protein involved in D-alaninealginate export/acetyltransferase of MBOAT family	dltB	3.649522	5.103430375
UniRef100_A0A1B9FNL6	Q8EZN4	EVE domain-containing protein	LA_3818	3.62578	3.181849842
UniRef100_A0A1B9FK57	Q8F432	Endoflagellar motor protein B	motB	3.599422	3.523472287

UniRef100_Q72R08	Q8F4S9	Protein translocase subunit SecA (EC 7.4.2.8)	secA	3.586422	4.493267961
UniRef100_A0A1X8WI23	Q8F706	Protein translocase subunit SecD	secD	3.577422	7.42354984
UniRef100_A0A1X8WMN9	Q8F3C8	Hypothetical lipoprotein	LA_2478	3.516691	2.75841534
UniRef100_Q72U12	Q8F142	Uridylate kinase (UK) (EC 2.7.4.22) (Uridine monophosphate kinase) (UMP kinase) (UMPK)	pyrH	3.516691	1.343171193
UniRef100_A0A868ASS0	Q8F0R6	Protein translocase subunit SecE	secE	3.484422	2.101581276
UniRef100_M6RK64	Q8F705	Protein-export membrane protein SecF	secF	3.431422	14.06601533
UniRef100_A0A0C5WR10	Q8F5I6	Protein-export membrane protein SecG	secG	3.407422	1.709431777
UniRef100_A0A1X8WM80	Q8F3C9	TfoX-like protein	tfoX	3.402178	3.140015821
UniRef100_A0A1B9FPW6	G1UB19	Protein translocase subunit SecY	secY	3.399422	3.191291013
UniRef100_A0A4D8S1L4	Q8F7E3	Virulence-associated protein VagC	vagC	3.398422	2.332041036
UniRef100_M6RPB3	Q8F539	ISLbp1 transposase	LA_1848	3.268716	2.095170989
UniRef100_A0A8G1DWE3	Q8F3I0	Chemotaxis response regulator CheY	LA_2423	3.189422	2.660358072
UniRef100_Q8F1S8	Q8F1S8	Hemolysin	LA_3050	3.048422	4.991701128
UniRef100_Q8F475	Q8F475	Lipoprotein with phospholipase D domain	LA_2170	3.043422	2.072847174
UniRef100_A0A1B9FQ95	Q8F8L7	Transcriptional regulator	LA_0538	3.037228	3.016174507
UniRef100_A0A0E2DBX6	Q8F492	Uncharacterized protein	LA_2150	-4.80558	2.187870857
UniRef100_A0A0E2CZS0	Q8F493	Uncharacterized protein	LA_2149	-4.80758	4.479171411
UniRef100_M3HKJ9	Q8F8Y3	SAM-dependent O-methyltransferase	LA_0415	-4.81101	6.746347349
UniRef100_Q72RH2	Q8F495	N-(5'-phosphoribosyl)anthranilate isomerase (PRAI) (EC 5.3.1.24)	trpF	-4.81358	3.388717397
UniRef100_Q8F496	Q8F496	DNA mismatch repair protein MutS	mutS	-4.83158	13.60103539

UniRef100_Q8F497	Q8F497	O-phosphoserine phosphohydrolase (EC 3.1.3.3)	serB	-4.85258	2.474391215
UniRef100_Q8F498	Q8F498	Biotin synthase (EC 2.8.1.6)	bioB	-4.85458	5.560438001
UniRef100_Q8F499	Q8F499	Adenosylmethionine-8-amino-7-oxononanoate aminotransferase (EC 2.6.1.62) (7,8-diamino-pelargonic acid aminotransferase) (DAPA AT) (DAPA aminotransferase) (7,8-diaminononanoate synthase) (DANS) (Diaminopelargonic acid synthase)	bioA	-4.89258	4.347484955
UniRef100_A0A8G0Z4K9	Q8EXZ5	Antagonist of anti-sigma factor	LB_062	-4.89265	11.54674266
UniRef100_Q72RG6	Q8F4A0	ATP-dependent dethiobiotin synthetase BioD (EC 6.3.3.3) (DTB synthetase) (DTBS) (Dethiobiotin synthase)	bioD	-4.90258	2.356386101
UniRef100_Q8F4A1	Q8F4A1	8-amino-7-oxononanoate synthase	bioF	-4.90458	2.261786364
UniRef100_P61975	Q8F4A2	Malate dehydrogenase (EC 1.1.1.37)	mdh	-4.90858	3.818044902
UniRef100_A0A8G0Z1S6	Q8F4A3	Oxidoreductase	dsbG	-4.91058	1.602550866
UniRef100_A0A1B9FHC5	Q8F4A4	Uncharacterized protein	LA_2137	-4.91858	8.489596171
UniRef100_A0A8G1E125	Q8F4A5	Uncharacterized protein	LA_2136	-4.92158	3.547527103
UniRef100_Q8EZH2	Q8EZH2	Putative lipoprotein	LA_3881	-4.93923	4.752396155
UniRef100_A0A868AV07	Q8F4A7	Homoserine kinase (HK) (HSK) (EC 2.7.1.39)	thrB	-4.94058	2.311334566
UniRef100_Q8F4A8	Q8F4A8	Sodium:solute symporter	putP	-4.94358	3.143229511
UniRef100_Q8F4A9	Q8F4A9	Putative hydrolase	LA_2132	-4.95858	3.621079299
UniRef100_A0A829CQU4	Q8F4B0	Uncharacterized protein	LA_2131	-4.98358	1.320936471
UniRef100_Q8F4B1	Q8F4B1	Aminotransferase (EC 2.6.1.-)	LA_2130	-5.01058	6.670254081
UniRef100_A0A1B9FHC7	Q8F4B2	DNA helicase (EC 3.6.4.12)	ssl2	-5.01158	5.56618918

UniRef100_Q8F4B4	Q8F4B4	Uncharacterized protein	LA_2127	-5.01358	5.242998093
UniRef100_A0A8G0Z577	Q8F4B6	Periplasmic serine protease	sppA	-5.02258	2.979363282
UniRef100_A0A4D8SDR0	Q8F4B7	Predicted membrane protein involved in D-alaninealginate export/acetyltransferase of MBOAT family	dltB	-5.03058	2.790687339
UniRef100_A0A0C5X8N8	Q8F9G8	Uncharacterized protein	LA_0227	-5.04107	6.211146134
UniRef100_A0A8G0Z3J5	Q8F4B8	Uncharacterized protein	LA_2123	-5.05058	2.419248524
UniRef100_M6KH81	Q8F9B6	Uncharacterized protein	LA_0279	-5.06672	4.276386235
UniRef100_A0A8G0Z2M4	Q8F4B9	Sigma factor regulatory protein	LA_2122	-5.07058	3.68701088
UniRef100_A0A1B9FLL1	Q8F4C0	Uncharacterized protein	LA_2121	-5.07458	5.949668457
UniRef100_Q8F4C1	Q8F4C1	Uncharacterized protein	LA_2120	-5.07758	5.169164252
UniRef100_A0A0E2CZJ2	Q8F4C2	Glycerol kinase	glpK	-5.08258	2.547928154
UniRef100_A0A1B9FLJ5	Q8F4C3	Metal-dependent hydrolase	LA_2118	-5.08358	14.92646526
UniRef100_A0A1B9FLL2	Q8F4C4	Anti-sigma factor antagonist	LA_2117	-5.10458	2.886759781
UniRef100_A0A0E2DC57	Q8F4C5	Zn-dependent hydrolase	gloB	-5.12058	1.873495424
UniRef100_A0A2H1XIS3	Q8F275	M23 family metalloendopeptidase	nlpD	-5.23084	2.646460987
UniRef100_P62642	Q8F5D8	Putative protein-glutamate methylesterase/protein-glutamine glutaminase (EC 3.1.1.61) (EC 3.5.1.44)	cheB2	-5.78258	2.468383984
UniRef100_A0A8G0YX99	Q8F2Z8	Flagellar motor switch protein FliN	LA_2613	-5.82658	6.308230066
UniRef100_A0A1B9FIQ3	Q8F3D7	Inhibitor of MCP methylation	LA_2469	-5.93058	6.56690727
UniRef100_Q72VP3	Q8F999	Zn-dependent alcohol dehydrogenase	adhP	-5.99058	5.045222235
UniRef100_A0A1B9FQ25	Q8F2D7	Adenylate/guanylate cyclase	cyaA	-6.01958	4.385869671
UniRef100_A0A8G1DYH2	Q8F9F3	Cytochrome c oxidase subunit 2 (EC 7.1.1.9)	cyoA	-6.02758	1.831796863
UniRef100_Q8F9F2	Q8F9F2	Cytochrome c oxidase subunit 1 (EC 7.1.1.9)	cyoB	-6.21958	11.14435393

UniRef100_P61443	P61443	Chaperone protein DnaK (HSP70) (Heat shock 70 kDa protein) (Heat shock protein 70)	dnaK	-6.50858	3.180013103
UniRef100_A0A8G1DVH4	Q8F3U1	Long-chain-fatty-acid CoA ligase	faa1	-6.64358	3.995171083
UniRef100_O51941	O51941	Flagellar filament 35 kDa core protein (35 kDa antigen) (Flagellin class B)	flaB	-6.83158	1.688090621
UniRef100_A0A8G1DZS1	Q8F4L7	Conserved hypothetical lipoprotein	LA_2024	-6.85758	3.357198691
UniRef100_M6RF76	Q8F4M3	Flagellin	LA_2017	-6.95558	2.000065619
UniRef100_A0A868AZC0	Q8F8Q0	Uncharacterized protein	LA_0505	-7.07458	2.170459058
UniRef100_Q72W52	Q8F9T9	Uncharacterized protein	LA_0100	-7.30758	3.679389258

Supplementary Table 4: (Proteomics supplementary file.xlsx)

As separate attachment

Supplementary Table 5: Top 50 Differentially expressed Leptospira proteins upon Host Interaction

**UniRef100**	**Uniprot ID**	**Protein names**	**Gene names (primary)**	**Log2_FC**	**minus_Log10_ pValue**
UniRef100_Q8F349	Q8F349	Glycosylhydrolase	bglX	5.91986443	12.0905008
UniRef100_Q72MQ7	Q8EZC7	Acyl-CoA dehydrogenase	caiA	5.364117141	16.49667926
UniRef100_Q8F6Q0	Q8F6Q0	Chemotaxis protein CheA (EC 2.7.13.3)	cheA	5.361487652	13.62547619
UniRef100_A0A0F6HG36	Q8F5D9	Methylase of chemotaxis methyl-accepting protein	cheR	5.324198552	16.49667926
UniRef100_A0A0E2CZ65	Q8F4N3	Flagellar motor switch protein FliG	fliG	5.272247193	11.63208642
UniRef100_A0A1X8WJY4	Q8F3H6	Chemotaxis protein CheW	cheW	5.272247193	9.246242244
UniRef100_M6RS94	Q8F0C1	Flagellar protein FliL	fliL	5.250923687	16.49667926
UniRef100_Q8EZS3	Q8EZS3	LigB-like protein	LA_3778	4.702435914	9.56017407
UniRef100_A0A0E2DJC8	Q8F7F0	Fibronectin type-III domain-containing protein	LA_0995	4.702435914	9.347559648
UniRef100_Q8F237	Q8F237	Uncharacterized protein	LA_2940	4.633256821	16.49667926
UniRef100_Q8F613	Q8F613	Cytoplasmic membrane protein	LA_1499	4.395611249	16.49667926
UniRef100_Q8F927	Q8F927	Uncharacterized protein	LA_0370	4.38259809	7.665536196
UniRef100_A0A0C5XH69	Q8F8R1	Putative lipoprotein	LA_0494	4.38259809	6.889046426
UniRef100_A0A1X8WI59	Q8F7R5	Uncharacterized protein	LA_0879	4.290645569	6.798085922
UniRef100_A0A8G0YXY6	Q8F8F9	Transcriptional regulator	LA_0598	4.181102551	6.116693526

UniRef100_A0A868AUI0	Q8F4G0	Flagellar motor switch protein FliM	fliM	4.130107179	7.001547213
UniRef100_A0A868AW11	Q8F3M5	Type II secretory pathway component protein C	gspC	4.023787614	16.49667926
UniRef100_Q8EXG7	Q8EXG7	Na+/solute symporter	putP	3.958657108	11.79491919
UniRef100_A0A8G0YXA1	Q8F3M6	Type II secretory pathway component protein D	gspD	3.958657108	7.226499706
UniRef100_Q8F3B8	Q8F3B8	Predicted membrane protein involved in D-alaninealginate export/acetyltransferase of MBOAT family	dltB	3.828428485	5.083842262
UniRef100_A0A1X8WNH3	Q8F3M7	Protein-secreting ATPase (EC 7.4.2.8)	gspE	3.755208065	5.338272479
UniRef100_A0A868AW95	Q8F3M8	Type II secretory pathway component protein F	gspF	3.630055939	8.598162369
UniRef100_A0A8G1DY50	Q8F3M9	Type II secretory pathway component protein G	gspG	3.600626577	16.49667926
UniRef100_Q72Q67	Q8F5Z7	RNA-binding protein Hfq	hfq	3.541267018	7.001547213
UniRef100_A0A0E2D7B3	Q8F808	TM2 domain-containing protein	LA_0782	3.514879713	7.580332294
UniRef100_Q8F3D6	Q8F3D6	Putative lipoprotein	LA_2470	3.355157256	5.001612594
UniRef100_A0A1B9FGS8	Q8F528	Catalase (EC 1.11.1.6)	katE	3.354028938	4.708829636
UniRef100_Q4PLB0	G1UB25	Outer membrane lipoprotein LipL21	lipL21	3.31019468	6.43756289
UniRef100_Q6J0P4	O34094	LipL32	lipL32	3.274261661	16.49667926
UniRef100_A0A1B9FPV7	Q8F0B9	Endoflagellar motor protein A	motA	3.222804561	7.162111825
UniRef100_M3HDK9	Q8F3V5	LipL45	lipL45	3.222804561	3.940952305
UniRef100_A0A8G0Z2K8	Q8F6B8	Uncharacterized protein	LA_1390	3.147795113	16.49667926
UniRef100_A0A0E2DGK7	Q8F3Z3	Predicted membrane protein involved in D-alaninealginate export/acetyltransferase of MBOAT family	dltB	3.147795113	5.776952083
UniRef100_A0A1B9FNL6	Q8EZN4	EVE domain-containing protein	LA_3818	3.111031312	5.594069111
UniRef100_A0A1B9FK57	Q8F432	Endoflagellar motor protein B	motB	3.0923766	16.49667926
UniRef100_Q72R08	Q8F4S9	Protein translocase subunit SecA (EC 7.4.2.8)	secA	3.036503334	16.49667926
UniRef100_A0A1X8WI23	Q8F706	Protein translocase subunit SecD	secD	3.036503334	5.660079238
UniRef100_A0A1X8WMN9	Q8F3C8	Hypothetical lipoprotein	LA_2478	2.933572638	13.62547619
UniRef100_Q72U12	Q8F142	Uridylate kinase (UK) (EC 2.7.4.22) (Uridine monophosphate kinase) (UMP kinase) (UMPK)	pyrH	2.902459533	7.139183036
UniRef100_A0A868ASS0	Q8F0R6	Protein translocase subunit SecE	secE	2.902459533	3.626473335
UniRef100_M6RK64	Q8F705	Protein-export membrane protein SecF	secF	2.598365205	5.107168279
UniRef100_A0A0C5WR10	Q8F5I6	Protein-export membrane protein SecG	secG	2.584000383	16.49667926
UniRef100_A0A1X8WM80	Q8F3C9	TfoX-like protein	tfoX	2.552868871	5.953161106
UniRef100_A0A1B9FPW6	G1UB19	Protein translocase subunit SecY	secY	2.552868871	4.31964352

UniRef100_A0A4D8S1L4	Q8F7E3	Virulence-associated protein VagC	vagC	2.341701164	2.972084165
UniRef100_M6RPB3	Q8F539	ISLbp1 transposase	LA_1848	2.287176752	2.323840106
UniRef100_A0A8G1DWE3	Q8F3I0	Chemotaxis response regulator CheY	LA_2423	2.183327706	3.558263636
UniRef100_Q8F1S8	Q8F1S8	Hemolysin	LA_3050	2.12035194	3.618586614
UniRef100_Q8F475	Q8F475	Lipoprotein with phospholipase D domain	LA_2170	2.068326861	2.59682172
UniRef100_Q8VTV7	G1UB31	Glycosyltransferase	LA_1642	-2.13289427	5.816917086
UniRef100_Q9AEE3	G1UB29	Glycosyl transferase	LA_1657	-2.13289427	5.001612594
UniRef100_A0A1X8WR29	Q8EZK5	Ig-like domain-containing protein	LA_3848	-2.13289427	2.292519364
UniRef100_Q8F4W3	Q8F4W3	Sec-independent protein translocase protein TatC	tatC	-2.139235797	4.860290101
UniRef100_A0A868ATW8	G1UB32	Serine protease	LA_2949	-2.139235797	2.114903318
UniRef100_Q9S4G0	G1UB33	Pyridoxal phosphate-dependent aminotransferase	wecE	-2.158429363	2.791369705
UniRef100_Q72W11	Q8F9P7	Probable GTP-binding protein EngB	engB	-2.158429363	2.174661156
UniRef100_Q9S4G1	G1UB34	FdtA-like protein	LA_1654	-2.184424571	3.674179273
UniRef100_A0A1B9FPR8	Q8F0B8	Flagellar FlbD family protein	LA_3578	-2.184424571	2.355684324
UniRef100_A0A1B9FI15	Q8F9F4	SCO1/SenC/PrrC family protein	LA_0241	-2.190997225	4.427529928
UniRef100_Q72VY9	O50638	Putative membrane protein insertion efficiency factor	LA_0177	-2.190997225	4.346021512
UniRef100_A0A1X8WTB8	G1UB35	Predicted transcriptional regulator	LA_2844	-2.190997225	3.561595912
UniRef100_Q72LU0	D4YW11	Uncharacterized protein	LA_4314a	-2.190997225	2.282593149
UniRef100_Q04NB1	P0C0A0	S-adenosylmethionine decarboxylase proenzyme (AdoMetDC) (SAMDC) (EC 4.1.1.50) [Cleaved into: S-adenosylmethionine decarboxylase beta chain; S-adenosylmethionine decarboxylase alpha chain]	speH	-2.19759996	4.868734303
UniRef100_A0A867ZJS6	Q8F847	Putative lipoprotein	LA_0712	-2.19759996	4.708829636
UniRef100_A0A868ATE4	Q8F2C6	Flagellar hook protein FlgE	flgE	-2.19759996	2.438915119
UniRef100_A0A0F6H573	Q8EYZ6	Two-component response regulator	LA_4065	-2.204233052	13.7482295
UniRef100_A0A1B9FG47	Q8F7X6	Response regulator containing a signal receiver domain and a DNA-binding domain	citB	-2.258425153	7.665536196
UniRef100_M3FQP6	Q8F7C7	NifU_N domain-containing protein	LA_1019	-2.258425153	4.024040006
UniRef100_M6K8Z5	Q8F1M9	LenB	LA_3103	-2.321928095	3.695034588
UniRef100_A0A1B9FIA4	Q8F301	Flagellar biosynthetic protein FliQ	fliQ	-2.351074441	2.84711932

UniRef100_Q8EZZ1	Q8EZZ1	Putative transcriptional regulator	LA_3710	-2.450084446	3.525799221
UniRef100_Q8F8S7	Q8F8S7	Tdh-like protein	LA_0477	-2.450084446	3.138988723
UniRef100_M6ZLK9	Q8EZE1	Putative lipoprotein	LA_3913	-2.506352666	7.596029532
UniRef100_Q72VL7	Q8F974	Fibronectin binding protein	LA_0322	-2.506352666	4.631886356
UniRef100_M7AD17	Q8EXV3	Transcriptional regulator	LB_104	-2.717856771	3.079645918
UniRef100_Q75FD8	Q8EX79	4a-hydroxytetrahydrobiopterin dehydratase (EC 4.2.1.96)	LB_335	-2.727379545	3.897189109
UniRef100_A0A0E2DB73	Q8EXP9	Precorrin-2 C-20 methyltransferase	cobF	-2.775959726	3.312386384
UniRef100_A0A1X8WR14	Q8F089	MaoC-like acyl dehydratase	maoC	-2.805912948	4.708787988
UniRef100_A0A0E2CYH1	Q8EZS1	Hypothetical lipoprotein	LA_3780	-2.805912948	3.98476052
UniRef100_M3HKJ9	Q8F8Y3	SAM-dependent O-methyltransferase	LA_0415	-2.816037165	7.997291409
UniRef100_A0A8G0Z4K9	Q8EXZ5	Antagonist of anti-sigma factor	LB_062	-2.867752202	3.828789486
UniRef100_Q8EZH2	Q8EZH2	Putative lipoprotein	LA_3881	-2.910501849	6.255747612
UniRef100_A0A0C5X8N8	Q8F9G8	Uncharacterized protein	LA_0227	-2.977099598	5.092612155
UniRef100_M6KH81	Q8F9B6	Uncharacterized protein	LA_0279	-3.058893689	7.726060219
UniRef100_A0A2H1XIS3	Q8F275	M23 family metalloendopeptidase	nlpD	-3.145605322	5.278480488
UniRef100_P62642	Q8F5D8	Putative protein-glutamate methylesterase/protein-glutamine glutaminase (EC 3.1.1.61) (EC 3.5.1.44)	cheB2	-3.224317298	5.775926854
UniRef100_A0A1B9FIQ3	Q8F3D7	Inhibitor of MCP methylation	LA_2469	-3.293358943	6.482088568
UniRef100_A0A8G0YX99	Q8F2Z8	Flagellar motor switch protein FliN	LA_2613	-3.293358943	5.88227517
UniRef100_Q72VP3	Q8F999	Zn-dependent alcohol dehydrogenase	adhP	-3.442222329	4.631886356
UniRef100_A0A1B9FQ25	Q8F2D7	Adenylate/guanylate cyclase	cyaA	-3.473931188	5.205511082
UniRef100_A0A8G1DYH2	Q8F9F3	Cytochrome c oxidase subunit 2 (EC 7.1.1.9)	cyoA	-3.506352666	6.634747244
UniRef100_P61443	P61443	Chaperone protein DnaK (HSP70) (Heat shock 70 kDa protein) (Heat shock protein 70)	dnaK	-3.60823228	7.726060219
UniRef100_Q8F9F2	Q8F9F2	Cytochrome c oxidase subunit 1 (EC 7.1.1.9)	cyoB	-3.60823228	7.332753152
UniRef100_A0A8G1DVH4	Q8F3U1	Long-chain-fatty-acid CoA ligase	faa1	-3.64385619	16.49667926
UniRef100_O51941	O51941	Flagellar filament 35 kDa core protein (35 kDa antigen) (Flagellin class B)	flaB	-3.680382066	16.49667926
UniRef100_A0A8G1DZS1	Q8F4L7	Conserved hypothetical lipoprotein	LA_2024	-3.836501268	16.49667926

UniRef100_A0A868AZC0	Q8F8Q0	Uncharacterized protein	LA_0505	-3.878321443	16.49667926
UniRef100_M6RF76	Q8F4M3	Flagellin	LA_2017	-3.878321443	4.620198364
UniRef100_Q72W52	Q8F9T9	Uncharacterized protein	LA_0100	-4.210896782	16.49667926

Supplementary Table 6: Topological parameters of PPI network determined by STRING v11.5 and Network analyser plugin of cytoscape 3.8.2

**STRING**	Number of nodes	130
	Number of edges:	162
	Average node degree:	2.49
	Avg. local clustering coefficient	0.361
	Expected number of edges:	88
	PPI enrichment p-value	1.21E-12
	Number of nodes (excluding isolated nodes)	70
**NETWORK ANALYZER**	Clustering coefficient	0.442
	Connected components	6
	Network diameter	7
	Network radius	4
	Shortest paths	470
	Characteristic path lengths	2.806
	Avg. number of neighbors	5.333
	Network density	0.13
	Network Heterogenecity	0.684
	Network Centrality	0.222

Supplementary Table S7: List of top 15% hub proteins identified in PPI network of 130 common DEGs and DEPs by consensus of betweenness and radiality statistical measures.

Protein names	Gene Name	Rank		Score	
		Betweenness	Radiality	Betweenness	Radiality
Capsule biosynthesis protein CapA	LA_1351	1	1	146.46	3.7756
secG	LA_1695	2	6	393.77	3.65
pyrH	LA_3296	3	8	349.31	3.64
nuoF	LA_0890	4	23	337.29	3.24
dnaK	LA_3705	5	5	251.41	3.67
purB	LA_3080	6	10	219.99	3.56

Uncharacterised protein	LA_0227	7	12	138.42	3.49
secA	LA_1960	8	2	122.86	3.731
lspA	LA_1336	9	9	120.86	3.6
ligB	LA_3778	10	25	93.78	3.23
fliM	LA_2081	11	43	93	0.98
Uncharacterised protein	LA_2900	12	26	80	3.21
rplJ	LA_3422	13	11	65.72	3.54
gspG	LA_2372	14	17	59.97	3.32
secY	LA_0759	15	3	49.19	3.72
secE	LA_3426	16	4	49.19	3.7
secD	LA_1142	20	7	33.25	3.64
tatC	LA_1925	25	13	29.26	3.39
gspC	LA_2376	30	14	19.47	3.35

Supplementary Table 8: 8 RNA-Protein pairs DEGs of *L. interrogans Icterohaemorrhagiae RGA* (*LEPIRGA*) at different times after interaction with the host. Gene expression correlation between qRT-PCR (THP1/HBPM/HWB) and RNA-Seq data

Protein ID	Description	2Hrs	6Hrs	12Hrs	24Hrs	RNA-Seq
**Q8EZX2**	Fimh-like protein (LA_3729)	4.01	-0.745	4.31	-1.915	1.727703
**Q8F613**	Cytoplasmic membrane protein (LA_1499)	-10.89	4.625	3.19	4.68	4.395611
**Q8F237**	Hypothetical protein (LA_2940)	-8.53	3.57	2.33	3.295	4.633257
**Q8F7F0**	Fibronectin type-III domain-containing protein (LA_0995)	-0.14	4.76	3.38	4.2	4.702436
**Q8EZH2**	Putative lipoprotein (LA_3881)	-3.305	-4.845	-5.995	-7.57	-2.9105
**Q8F974**	Fibronectin binding protein (LA_0322)	-11.055	-11.5	-13.83	-13.58	-2.50635
**Q8F1M9**	LenB (LA_3103)	-7.42	-8.89	-10.575	-10.185	-2.32193
**Q8F2C6**	Flagellar hook protein FlgE (LA_2848)	-7.705	-9.9	-11.935	-11.725	-2.1976
**O34094**	Lipl32 (LA_2637)	6.05	3.255	2.25	2.305	3.769

Supplementary Table 9: Primer sequence of selected 8 genes for validation

Uniprot Id	Gene name	Primer Sequence (5' – 3')	Amplicon Length (bp)
**Q8EZX2**	Fimh-like protein	AAATTAGAAAGTTATATG	138
		GCCAAATCAGCCCCGTTC	
**Q8F613**	cytoplasmic membrane protein	GCATATTTAGATTTTAGC	143
		TGGATACTAGAAGAATCC	
**Q8F237**	hypothetical protein	GATTCTGGATGGATAATC	130
		CATAAGTCAGTCTTGAAC	
**Q8F7F0**	Fibronectin type-III domain-containing protein	GAAGAAAGAAAGTATACC	134
		TCTAAACTACTAGTGACC	

**Q8EZH2**	putative lipoprotein	CAGAGAATTATAAAAACC	136
		CAATCGCAAAGGAACTTC	
**Q8F974**	fibronectin binding protein	TCATTTCAAAATTCTTCC	137
		GTAAGAGGAACCGTTTGG	
**Q8F1M9**	LenB	ATGGGTACAAGAGAAGCG	138
		AGTTACAACTGAGTTTGC	
**Q8F2C6**	flagellar hook protein FlgE	ATGAGGTCACTCTATTCC	140
		TCCTGAGAAATCATATCC	
	16S	TAAAGGCTCACCAAGGCGAC	198
		TTAGCCGGTGCTTTAGGCAG	
**O34094**	Lipl32	AAGCATTACCGCTTGTGGTG	242
		GAACTCCCATTTCAGCGATT	
